# Coordinated regulation of *Arabidopsis* microRNA biogenesis and red light signaling through Dicer-like 1 and phytochrome-interacting factor 4

**DOI:** 10.1371/journal.pgen.1007247

**Published:** 2018-03-09

**Authors:** Zhenfei Sun, Min Li, Ying Zhou, Tongtong Guo, Yin Liu, Hui Zhang, Yuda Fang

**Affiliations:** National key Laboratory of Plant Molecular Genetics, CAS Center for Excellence in Molecular Plant Sciences, Institute of Plant Physiology and Ecology, Chinese Academy of Sciences, Shanghai, China; University of the Chinese Academy of Sciences, Beijing, China; Peking University, CHINA

## Abstract

Light and microRNAs (miRNAs) are key external and internal signals for plant development, respectively. However, the relationship between the light signaling and miRNA biogenesis pathways remains unknown. Here we found that miRNA processer proteins DCL1 and HYL1 interact with a basic helix-loop-helix (bHLH) transcription factor, phytochrome-interacting factor 4 (PIF4), which mediates the destabilization of DCL1 during dark-to-red-light transition. PIF4 acts as a transcription factor for some miRNA genes and is necessary for the proper accumulation of miRNAs. DCL1, HYL1, and mature miRNAs play roles in the regulation of plant hypocotyl growth. These results uncovered a previously unknown crosstalk between miRNA biogenesis and red light signaling through the PIF4-dependent regulation of miRNA transcription and processing to affect red-light-directed plant photomorphogenesis.

## Author summary

External light and internal miRNAs are important for plant development. This study revealed that the miRNA-processing enzyme DCL1 interacts with the red-light-regulated transcription factor PIF4, which modulates the stability of DCL1 during dark-to-red-light or red-light-to-dark transitions and acts as a transcription factor for some miRNA genes. This study revealed that DCL1 and mature miRNAs play roles in the red light signaling pathway to regulate plant photomorphogenesis. These results shed light on the crosstalk between miRNA and red light signaling pathways.

## Introduction

Light is one of the most important environmental factors to regulate multiple growth and developmental processes of plants, including germination, de-etiolation, phototropism, flowering, leaf and stem growth, circadian clock adjustment, stomatal opening, chloroplast relocation, and anthocyanin synthesis [1,2]. Plants utilize at least four distinct families of photoreceptors, including phytochromes, cryptochromes, phototropins, and the ultraviolet B photoreceptor, to perceive light signals [3,4]. Phytochromes (phys) are primarily responsible for detecting red and far-red light. The *Arabidopsis* genome encodes five phys, phyA to E [3]. Of these, phyA and phyB have the most prominent functions: phyA is responsible for perceiving far-red (FRc) light, and phyB for continuous monochromatic-red (Rc) light [5]. Members of the basic helix-loop-helix (bHLH) family of transcription factors play a central role in phytochrome-mediated signal transduction. Among bHLH factors, phytochrome-interacting factor 4 (PIF4) acts as a negative regulator in the phyB signaling pathway by selectively binding to the biologically active Pfr form of phyB and regulating a subset of downstream genes [6]. It also acts as a mediator in the auxin-signaling pathway at high temperature, playing a key role in modulating developmental responses to both light and temperature [7]. In addition, PIF4 integrates the brassinosteroid (BR) and light signals by interacting with BZR1 and binding to nearly two thousand common target genes, and synergistically regulating many of these target genes [8]. Recently, it has been reported that PIF4 interacts with cryptochrome 1(CRY1) to regulate high temperature-mediated hypocotyl elongation under blue light [9].

The 20–22 nt-long miRNAs are essential regulators for many biological processes in almost all eukaryotes [10]. MiRNAs are processed from long stem-loop primary transcripts (pri-miRNAs), which are transcribed by DNA-dependent RNA polymeraseII [11]. In animals, the pri-miRNAs are first cropped in the nucleus by the RNAse-III-like endonuclease Drosha and its partner DGCR8, a double-stranded RNA (dsRNA) binding (dsRBD) protein, to release the foldback precursor miRNAs (pre-miRNAs). After exportin-5-mediated export to the cytoplasm, the pre-miRNAs are cut into the miRNA/miRNA* duplexes by the Drosha homolog Dicer with the assistance of TAR RNA-binding protein 2 (TRBP) [12,13]. In plants, however, the two processing steps are completed in the nucleus by a single RNase-III enzyme, DICER-LIKE1 (DCL1) [14,15]. Other proteins involved include the dsRBD protein, HYPONASTIC LEAVES1 (HYL1) [16], and the zinc finger domain protein, serrate (SE) [17,18]. In addition, several transcription factors including CDF2, CDC5, MeCP2, and NOT2 facilitate miRNA processing [19–22]. Recently, the regulatory mechanism of miRNA processor stability has been partially revealed. In plants, HYL1 is modulated by the light signaling factor COP1, and destabilized by an unidentified protease [23]. In animals, Dicer is degraded through autophagy [24].

In this study, we show that DCL1 interacts with PIF4 which integrates miRNA biogenesis and red light signaling by regulating the transcription of a group of miRNA genes and the stability of DCL1 during dark-to-red-light or red-light-to-dark transitions. Our results also revealed a previously unknown role for the miRNA processing enzyme DCL1 in red light signaling.

## Results

### DCL1 interacts with PIF4

**Fig 1:**
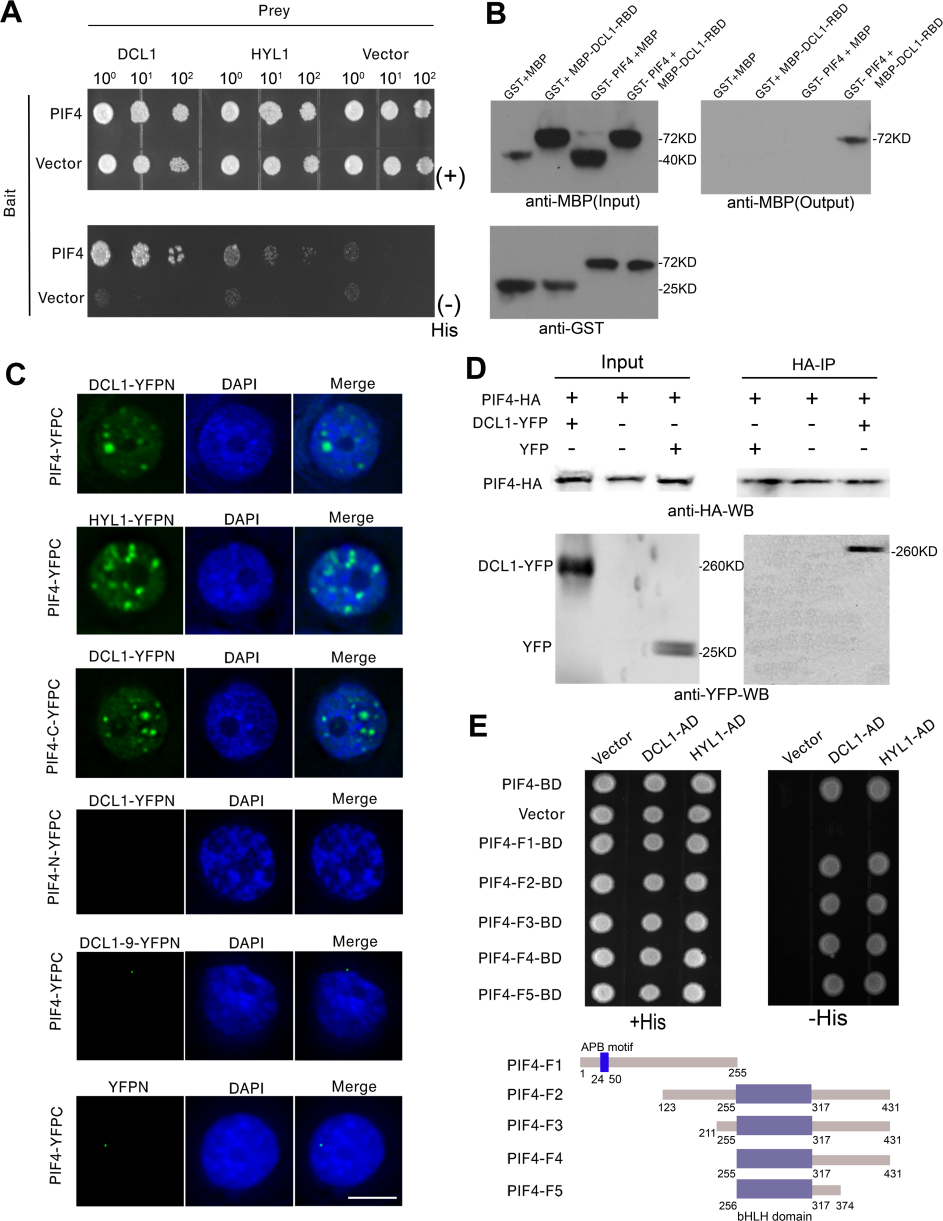
DCL1 interacts with PIF4. **(A)** Yeast two-hybrid assays show the interactions between PIF4 and DCL1, or PIF4 and HYL1. Co-transformed yeast colonies were spotted on the selective SC medium minus Trp and Leu, then grown on SC medium minus His, Trp, and Leu supplemented with 5mM 3-amino-1, 2, 4-triazole (3-AT). **(B)** MBP pull-down assay shows the interaction between PIF4 and the double strand RNA binding domains (RBD) of DCL1. *In vitro* pull-down assay using *E*. *coli*–expressed MBP, MBP-DCL1-RBD, GST and GST-PIF4. GST- PIF4 was incubated with MBP-DCL1-RBD bound to amylose resin. The upper panel (input): an anti-GST antibody was used to detect GST or GST- PIF4. The bottom panel (pull-down): an anti-MBP antibody was used to detect MBP-DCL1-RBD. **(C)** BiFC assays show the interaction between PIF4 or the C-terminal fragment of PIF4 (PIF4-C) and DCL1or HYL1 in tobacco epidermal cells. No interactions were detected between PIF4 and YFP, PIF4 and DCL1-9, or the N-terminal fragment of PIF4 (PIF4-N) and DCL1. (Scale bar = 10 μm). **(D)** Co-IP assay shows the interaction between PIF4 and DCL1. *Arabidopsis* plants were transformed with *pPIF4*::*PIF4-HA* and *pDCL1*::*DCL1-YFP*. The extracts from 4-day-old seedlings were incubated with anti-HA–conjugated agarose. The pellet was analyzed by immunoblotting with anti-HA and anti-GFP antibodies. **(E)** Yeast two-hybrid assays show that the C-terminal fragments of PIF4 interact with DCL1-RBD. Diagrams of the PIF4 fragments (F1-F4) and domains within F1-F4 are shown at the bottom panel: F1, aa 1–255; F2, aa 123–431; F3, aa 211–431; F4, aa 255–431; F5, aa 256–374; APB, active phytochrome binding motif; bHLH domain, basic helix-loop-helix domain.

To study the function of DCL1, we performed yeast two-hybrid screens to identify proteins that interact with the two C-terminal DsRBDs of DCL1 (DCL1-RBD) which are important for protein–protein interaction [25]. In addition to the transcription factor CDF2 [21], we obtained another transcription factor, PIF4. We then examined the interactions between PIF4 and full-length DCL1 or HYL1 by yeast two-hybrid assays. Our results show that PIF4 can interact with DCL1 and HYL1, the essential components in miRNA processing (Fig 1A).

We confirmed the interaction between DCL1-RBD and PIF4 by MBP pull-down assays. The fusion proteins MBP-DCL1-RBD and GST-PIF4 were expressed in *E*. *coli* and purified with amylose resin and glutathione sepharose beads, respectively. We incubated GST-PIF4 with the MBP-DCL1-RBD captured by amylose resin beads (Fig 1B). Parallel assays were performed using GST and MBP proteins as negative controls (Fig 1B). The results indicated that MBP-DCL1-RBD can interact with GST-PIF4, whereas no interaction was observed in the negative controls (Fig 1B).

To verify the interaction between DCL1 and PIF4 *in vivo*, we performed the bimolecular fluorescence complementation (BiFC) assay. We fused DCL1 to the N-terminal fragment of yellow fluorescent protein, YFP (YFPN), and PIF4 to the C-terminal fragment of YFP (YFPC). The fusion pairs were transiently co-transformed into tobacco leaf epidermal cells by *Agrobacterium*-mediated infiltrations. Strong BiFC signals were detected in nuclear bodies for DCL1 and PIF4, HYL1 and PIF4, and DCL1 and the C-terminal fragment of PIF4 (PIF4-C) (Fig 1C). In contrast, we did not observe any BiFC signals between the N-terminal fragment of PIF4 (PIF4-N) and DCL1, PIF4 and YFP, or PIF4 and DCL1-9, a C-terminal DsRBD-truncated form of DCL1 from 1733aa-1911aa [26].

We performed coimmunoprecipitation (Co-IP) experiments to further confirm the interaction *in vivo*. We extracted total proteins from the 4-day-old seedlings of *Arabidopsis* plants transformed with *pPIF4*::*PIF4*-HA and *pDCL1*::*DCL1*-YFP [21](S1 Fig), and we extracted the proteins from plants co-expressing *pPIF4*::*PIF4*-HA and *p35S*::*YFP* as a negative control. We incubated the extracted proteins with HA-conjugated agarose beads to immunoprecipitate DCL1. We separated DCL1-containing complexes using SDS-PAGE and immunoblotted them with anti-GFP and anti-HA antibodies. Fig 1D shows the physical interaction between PIF4 and DCL1.

PIF4 has an N-terminal active phytochrome binding(APB) motif [27] and a C-terminal bHLH domain [28]. We mapped the region of PIF4 that interacts with DCL1 by yeast two-hybrid assays. We found that the C-terminal region of PIF4 interacts with DCL1 as strongly as the full-length PIF4 (Fig 1E), and this was consistent with the BiFC results (Fig 1C). The transcriptional activation activity of the C-terminal region of PIF4 is shown in S2 Fig.

### PIF4 promotes the accumulation of DCL1 during red-light-to-dark transition

**Fig 2:**
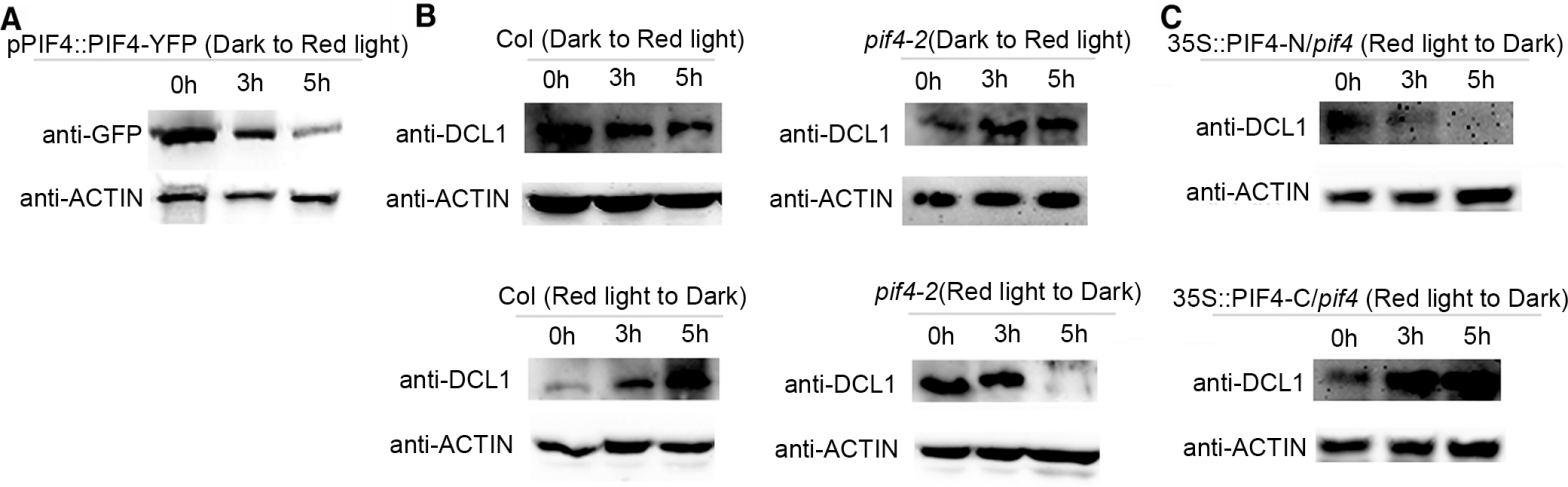
DCL1 is destabilized by PIF4 during dark to red light transition. **(A)** Anti-GFP western blots of *pPIF4*::*PIF4-YFP*/Col seedlings grown in continuous darkness for 4 d and then transferred to red light condition for 3 or 5 h. **(B)** Anti-DCL1 western blots of WT and *pif4-2* mutant seedlings grown in red light for 4 d followed by transferring to dark condition for 3 or 5 h (red light to dark), or grown in continuous darkness for 4 d followed by transferring to red light for 3 or 5 h (dark to red light). **(C)** Anti-DCL1 western blots of *p35S*::*PIF4-N*/*pif4-2*, *p35S*::*PIF4-C*/*pif4-2* seedlings grown in red light for 4 d and then transferred to dark condition for 3 and 5 h. All the western blots have been performed for three biological replicates.

PIF4 is regulated by red light [17,18], and the accumulation level of PIF4 protein decreases during the transition from darkness to red light (Fig 2A, S3A Fig, S4A Fig). We examined whether the accumulation level of DCL1 changes during red-light-to-dark and dark-to-red-light transitions. Four-day-old wild-type (WT) seedlings were grown in continuous red light or darkness and then transferred to the opposite light condition for 3 and 5 h. Western blots of the protein extracts from these seedlings revealed that the DCL1 protein level decreases during dark-to-red-light transition (Fig 2B, S3B Fig, S4B Fig), and increases during red-light-to-dark transition at 3 and 5 h (Fig 2B, S3B Fig, S4B Fig).

To further address the stability of DCL1 protein *in vivo*, we generated *pDCL1*::*DCL1-YFP*/Col lines and the protein accumulation level of DCL1-YFP was monitored by Western blots using an anti-GFP antibody. We found that red light promotes the destabilization of DCL1 (S5 Fig).

We then compared DCL1 protein levels in WT seedlings and *pif4-2* mutant seedlings in response to red light for 3 and 5 h, and found that the DCL1 protein level gradually decreases in the *pif4-2* mutant during red-light-to-dark transition, but increases during dark-to-red-light transition at 3 and 5 h (Fig 2B, S3B Fig, S4B Fig). These results suggested that PIF4 promotes the destabilization of DCL1 during dark-to-red-light transition at 3 and 5 h.

As the C-terminal region of PIF4 interacts with DCL1 and has transcriptional activation activity, we examined the DCL1 levels in two transgenic lines expressing N-terminal (PIF4-N) or C-terminal (PIF4-C) protein fragment of PIF4 in the *pif4-2* mutant background (S1 Fig). The results show that DCL1 protein levels in *35S*::*PIF4-N*/*pif4-2* transgenic lines were similar to those in the *pif4-2* mutant (Fig 2B and 2C, S3B and S3C Fig, S4B and S4C Fig). In contrast, the DCL1 levels in *35S*::*PIF4-C*/*pif4-2* transgenic lines were similar to those in the WT seedlings (Fig 2B and 2C, S3B and S3C Fig, S4B and S4C Fig). These results indicated that the C-terminal fragment of PIF4 can rescue the DCL1 destabilization in the *pif4*-2 mutant during red-light-to-dark transition.

We then examined the accumulation level of HYL1 and tested the effect of PIF4 on HYL1 stability by comparing HYL1 levels in Col and *pif4* mutant backgrounds during red-light-to-dark or dark-to-red-light transitions. The results showed that the HYL1 protein level decreases during dark-to-red-light transition and increases during red-light-to-dark transition at 3 and 5 h in the WT seedlings (S6 Fig). In contrast, the HYL1 protein level increases during dark-to-red-light transition and decreases during red-light-to-dark transition at 3 and 5 h in the *pif4* mutant (S6 Fig), indicating that PIF4 promotes the destabilization of HYL1 during dark-to-red-light transition.

To elucidate whether the expressions of *DCL1* and *HYL1* were modulated at the mRNA level under red light, we tested the *DCL1* and *HYL1* transcript levels in WT and *pif4-2* seedlings by real time quantitative PCR (qRT-PCR). We found that the *DCL1* and *HYL1* transcript levels in *pif4-2* are similar to those of corresponding genes in WT under red light (S7A and S7B Fig), indicating that the accumulation levels of DCL1 and HYL1 proteins under red light is mainly regulated at the post-transcriptional level.

### DCL1 is degraded through a pathway independent of ubiquitin-proteasome

**Fig 3:**
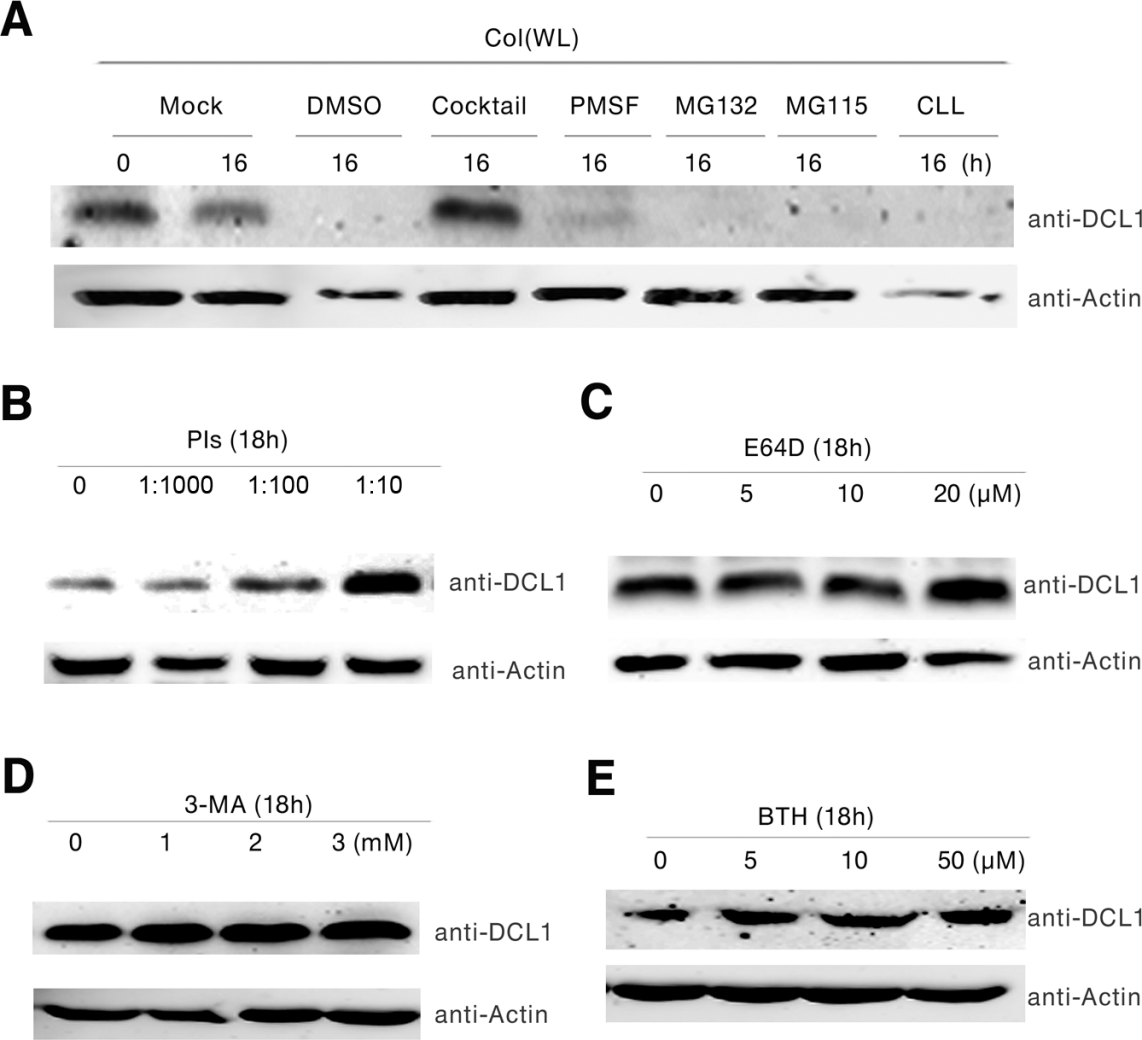
DCL1 is not degraded through ubiquitin proteasome pathway. **(A)** DCL1 protein levels in WT seedlings in the presence of different inhibitors. Extracts were treated with nothing (-), 2% DMSO (solvent for the inhibitors), 40 μM PIs, 4 mM PMSF, 40 μM MG132, MG115 or CLL. After 2 h of incubation at room temperature, the DCL1 levels were detected by western blots. ACTIN was used as a loading control. **(B)** Western blot analysis of seedlings incubated with PIs (1:1000, 1:100, and 1:10 dilutions). **(C)** Western blot analysis of seedlings incubated with E64D (0–20 mM). **(D)** Western blot analysis of seedlings incubated with 3-MA (0–2 mM). **(E)** Western blot analysis of seedlings incubated with BTH (0–50 mM). The levels of DCL1 were determined with an anti-DCL1 antibody. Equal loading of samples was determined by an anti-ACTIN antibody. All the western blots have been performed for three biological replicates.

Since the ubiquitin-proteasome (UPS) and autophagy are two major proteolytic pathways for protein degradation in plants [29,30], we decided to test whether the instability of DCL1 protein is regulated by these two pathways. First, we applied several proteolysis inhibitors, including MG132 (a reversible proteasome inhibitor), MG115 (a proteasome-specific inhibitor), CLL (clasto-lactacystin b-lactone, a specific irreversible proteasome inhibitor in UPS), proteolysis inhibitors (PIs) (cocktail, a protease inhibitor mixture), PMSF (phenylmethylsulfonyl fluoride, a serine protease inhibitor) to examine whether proteolysis is responsible for DCL1 stability, using the solvent dimethyl sulfoxide (DMSO) as a negative control. We found that only the broad-spectrum PIs stabilize the DCL1 protein, whereas the proteasome-specific inhibitors MG132, MG115, and CLL have no stabilizing effect on the protein (Fig 3A). These data indicated that the destabilization of DCL1 is not driven by the UPS pathway.

To investigate whether the autophagic pathway regulates the proteolysis of DCL1, we treated 4-day-old WT seedlings for 18h with several protease inhibitors, including PIs and E64D (an irreversible cysteine protease inhibitor which can strongly inhibit autophagic degradation). As shown in Fig 3B and 3C, the prolonged treatments of these inhibitors at high concentrations could increase the DCL1 protein level. Given that the autophagic pathway is composed of three steps: autophagopore formation; substrate loading into the autophagosome; and transition of autophagosome into vacuoles for substrate degradation, we treated WT seedlings for 18 h with 3-MA, an inhibitor of type III phosphatidylinositol 3-kinases that blocks the formation of autophagosomes, or BTH, a salicylic acid analog that stimulates the formation of autophagosomes, to determine if these autophagic processes are involved in the control of DCL1 levels. We found only a mild change in the DCL1 protein levels under either of the 3-MA and BTH treatments (Fig 3D and 3E), suggesting that further studies are necessary to uncover if and how the DCL1 protein undergoes autophagy during dark to red light transition.

### PIF4 affects the accumulation of a group of miRNAs

As red light modulates DCL1 stability, we applied high-throughput sequencing using WT seedlings grown in dark for 4 days and then exposed to continuous red light for 2 h and 8 h to determine the global effects of red light on miRNA accumulation. The sequencing data for all known miRNAs were subjected to hierarchical clustering in an unsupervised manner [31] to analyze the expression of 2-fold differential miRNAs among plants held under red light for 2 h and 8 h compared with their miRNA levels at 0 h (S8 Fig). The results show that the expression levels of many miRNAs changed under 2 h and 8 h red light treatments.

**Fig 4:**
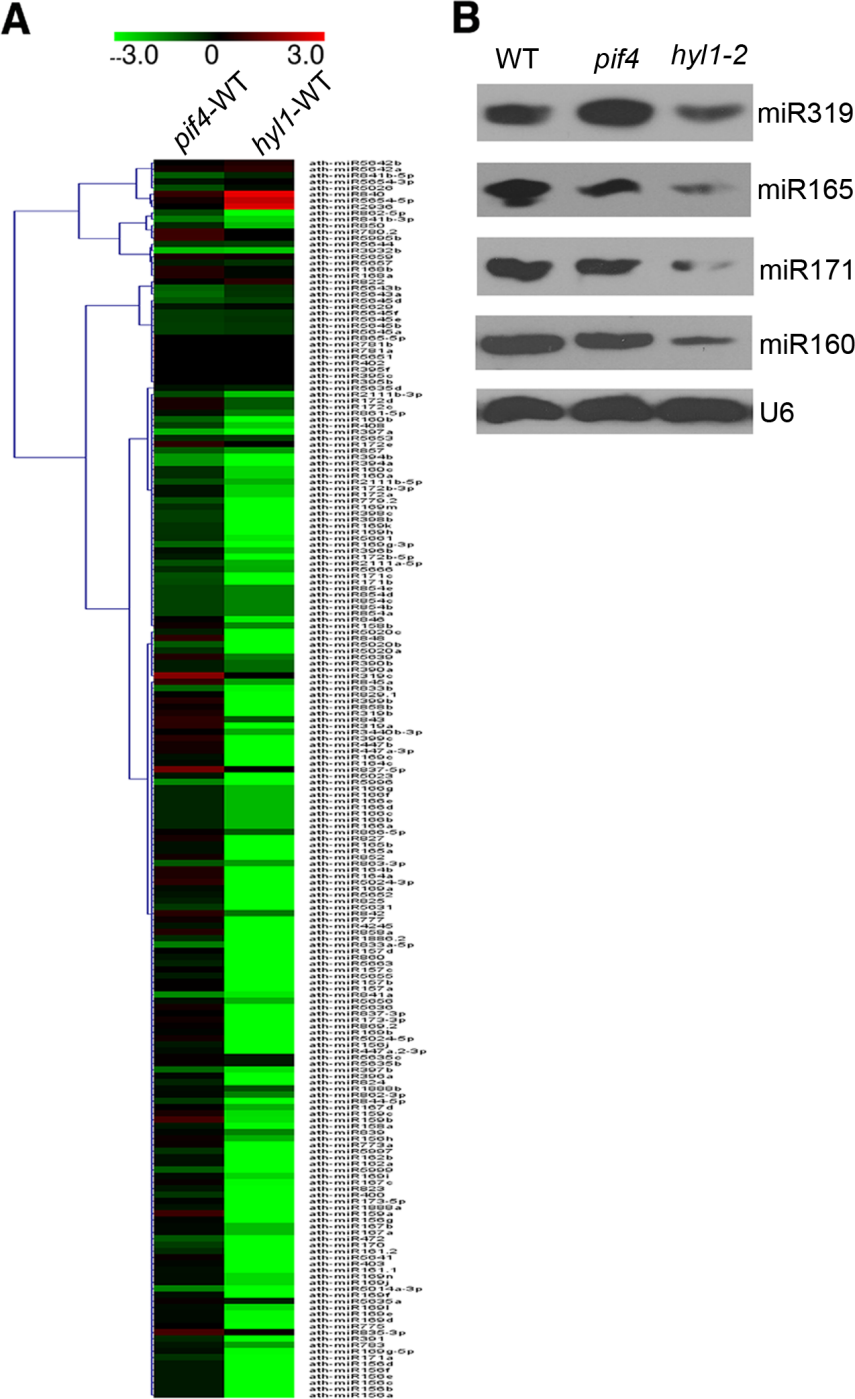
PIF4 is required for the proper accumulation of miRNAs. **(A)** Heat map of miRNAs differentially expressed in *pif4-2* (WT—*pif4-2*) and *hyl1-2* (WT—*hyl1-2*) mutants compared to WT. Four-day old red light grown seedlings were collected for RNA extraction. Small RNAs were isolated and sequenced by Solexa high-throughput sequencing. **(B)** Northern blots show the miRNA levels in seedlings of WT, *pif4-2* and *hyl1-2* grown for 4 days under red light (10 μmolm^-2^s^-1^). U6 serves as a loading control.

As PIF4 interacts with DCL1 and affects its stability, we analyzed the global miRNAs in 4-day-old WT seedlings and *pif4-2* mutant seedlings under continuous red light to determine the effect of PIF4 on miRNA accumulation, using *hyl1* mutant seedlings as a control. The data quality is summarized in S1 and S2 Tables. The sequencing data for all known miRNAs were subjected to hierarchical clustering in an unsupervised manner to analyze the expression of 2-fold differential miRNAs [31] (Fig 4A and S9 Fig). The small RNA-seq data chosen from the changed miRNAs were validated by Northern blots of miR319, miR165, miR171, and miR160 (Fig 4B). Among 189 miRNAs detected in both WT and *pif4-2* mutant seedlings, we found that 22 miRNAs show at least 1.5-fold changes in the *pif4-2* mutant compared with those in the WT. Among these 22 miRNAs, 21 (95.5%) were down-regulated, and one (4.5%) was up-regulated. Taking these observations together, we concluded that the accumulation of a group of miRNAs is affected by PIF4.

### PIF4 is a transcription factor for a population of miRNA genes

**Fig 5:**
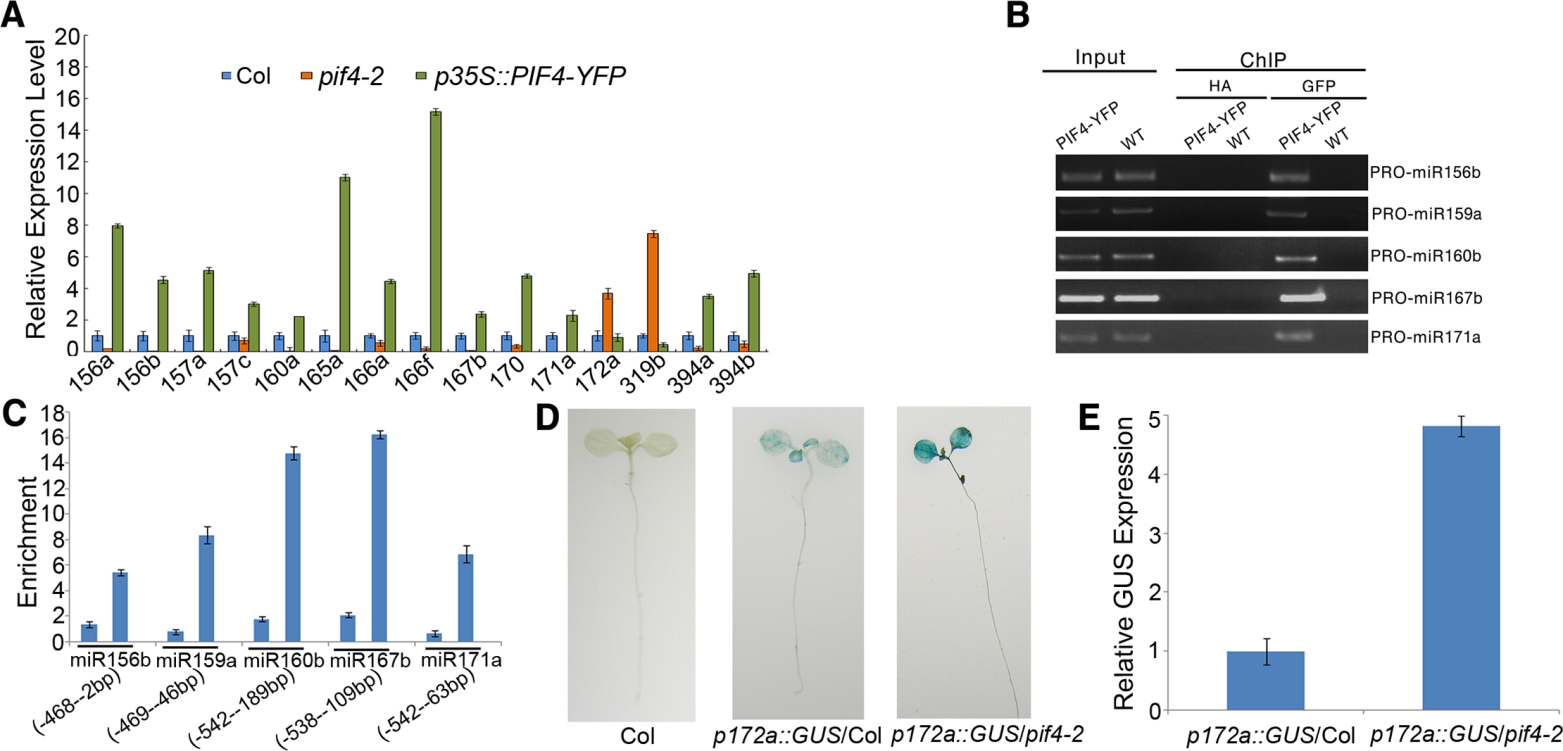
PIF4 binds to the promoters of miRNAs genes to regulate their expressions. **(A)** The relative expression levels of pri-miRNAs in *pif4-2* and *35S*::*PIF4-YFP* by real-time PCR compared to those in WT. The relative fold changes were normalized to *ACTIN*. Data are given as means ± SD (n = 3 biological replicates). **(B)** ChIP-PCR analysis of five promoter fragments of miRNA genes in WT and *pPIF4*::*PIF4-YFP* seedlings. ChIP assays were performed using the 4-d-old WT and *pPIF4*::*PIF4-YFP* seedlings. DNA was amplified using primers specific to five miRNA promoter regions. **(C)** ChIP followed by real time PCR of five promoter fragments of 5 miRNA genes in WT and *pPIF4*::*PIF4-YFP* seedlings, the start and stop positions of the amplified fragments were shown in brackets under each miRNA. Relative enrichment of fragments was calculated with the HA antibody as a control. For each miRNA, the left column represents anti-HA sample and the right column represents anti-GFP sample. Data are given as means ± SD. All the experiments have been performed for three biological replicates. **(D)** Histochemical GUS staining of WT, *pMIR172a*::*GUS/*WT and *pMIR172a*::*GUS/pif4-2* seedlings. Thirty WT plants or plants harboring a *GUS* gene were analyzed for these genotypes. **(E)** qRT-PCR analysis of the transcript levels of *GUS* driven by miR172a promoter in WT and *pif4-2* seedlings. Data are given as means ± SD. All the experiments have been performed for three biological replicates.

In the sequencing data, we noticed that the levels of some miRNAs decrease in the *pif4-2* mutant in which the DCL1 accumulation level increases, so we speculated that PIF4 may play a role in miRNA transcription. As PIF4 is a transcription factor in the red light signaling pathway, we analyzed the expression levels of 14 pri-miRNAs, for which their mature miRNAs show differences in the *pif4* mutant compared with those in the WT (S1 Table), by qRT-PCR in seedlings of *pif4-2* mutant and *PIF4* overexpression (*p35S*::*PIF4-YFP*) lines. As shown in Fig 5A, the relative expression levels of pri-miRNAs between *pif4-2* and *p35S*::*PIF4-YFP* were predominantly opposite for all 14 pri-miRNAs, although some of pri-miRNAs were up-regulated, whereas others were down-regulated in the *pif4-2* mutant line (Fig 5A), indicating that PIF4 might act as a transcription factor for these miRNA genes.

To address whether PIF4 binds to the promoters of miRNA genes, we performed chromatin immunoprecipitation-PCR (ChIP-PCR) using GFP antibody-precipitated chromatin from *pPIF4*::*PIF4-YFP*/Col plants. We focused on the promoters of miRNA genes for which the expression levels of pri-miRNAs were found to have changed in the *pif4-2* mutant (Fig 5A). The promoter fragments of miRNA genes were amplified from GFP antibody-immunoprecipitated, but not from HA antibody-immunoprecipitated *pPIF4*::*PIF4-YFP*/Col samples. In addition, no apparent enrichment of fragments in the WT seedlings was observed (Fig 5B and 5C). We performed DNA competitive electrophoretic mobility shift assays to verify the direct interaction between PIF4 and a fragment of miR160b promoter containing a G-box DNA-sequence motif (CACGTG), which is similar to the E-box motif (CANNTG) known to be the binding site of PIFs [32]. The reactions were performed using decreasing amounts of PIF4, and the results showed that PIF4 can directly bind to the promoter of miR160b gene (S10 Fig). We concluded that PIF4 is a transcription factor for a group of miRNA genes.

To further test the effect of PIF4 on miRNA expression, we used a β-glucuronidase (*GUS*) reporter gene driven by the promoter of *miR172a* whose expression is repressed by PIF4 (Fig 5D). This system was previously used to determine the function of CDF2, DDL, CDC5, and NOT2 in the regulation of miRNA gene transcription [19–21,33]. We crossed *pif4-2* mutant with transgenic plants containing *pmiR172a*::*GUS*. In the second (F_2_) generation, we obtained *PIF4*/*PIF4*, *PIF4*/*pif4*, and *pif4/pif4* genotypes containing *pMIR172a*::*GUS*. The expression level of *GUS* increases in *pif4/pif4* compared with that in *PIF4*+ plants (Fig 5D). Quantitative RT-PCR analysis indicated that the *GUS* level in the *pif4* mutant increases compared with that in WT plants (Fig 5E). We concluded that PIF4 regulates the transcription of *miR172a* gene.

### DCL1 and HYL1 negatively regulate plant photomorphogenesis

**Fig 6:**
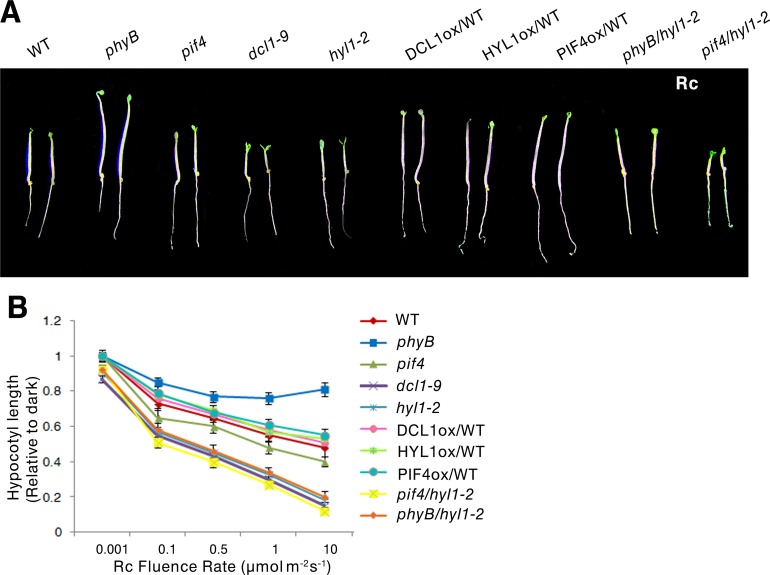
The roles of PIF4, DCL1 and HYL1 in plant photomorphogenesis. **(A)** Visual phenotypes of WT, *phyB*, *pif4-2*, *dcl1-9*, *hyl1-2*, *p35S*::*DCL1-YFP*/Col, *p35S*::*HYL1-YFP*/Col, *p35S*::*PIF4-YFP*/Col and *phyB*/*hyl1-2*, *pif4-2*/*hyl1-2* seedlings grown under red light (10 μmolm^-2^s^-1^) for 4 days. **(B)** Rc fluence rate response curves for hypocotyl lengths of WT, *phyB*, *pif4-2*, *dcl1-9*, *hyl1-2*, *p35S*::*DCL1-YFP*/Col, *p35S*::*HYL1-YFP*/Col, *p35S*::*PIF4-YFP*/Col, *phyB*/*hyl1-2*, and *pif4-2*/*hyl1-2* seedlings grown under red light (10 μmolm^-2^s^-1^) for 4 days. Data are means ± SEM of 30 plants. All the experiments have been performed for three biological replicates.

As PIF4 interacts with red light receptor phyB [6] and miRNA processing enzyme DCL1 (Fig 1), we investigated the role of DCL1 in photomorphogenesis by examining the light inhibition of hypocotyl elongation, the most widely used phenotype to study photomorphogenesis [34]. We compared the hypocotyl growth of 4-day-old WT seedlings and *pif4-2*, *hyl1-2*, and *dcl1-9* mutant seedlings grown under continuous red light at four different intensities (0.1, 0.5, 1,and 10 μmol s^−1^m^−2^), or continuous darkness. The hypocotyl lengths of the mutants were indistinguishable with those of the WT grown under dark condition; only slightly shorter hypocotyls were observed in *hyl1-2* and *dcl1-9* mutants grown under dark condition (S11A and S11B Fig). The relative hypocotyl lengths under red light were normalized to the hypocotyl lengths of dark-grown seedlings [35], and analyses indicated that *dcl1-9* and *hyl1-2* had shorter hypocotyl lengths than WT seedlings (Fig 6A and 6B). To diminish the developmental effects of *dcl1-9* and *hyl1-2* mutants on the analysis of photomorphogenesis based on hypocotyl lengths, we examined the hypocotyl growth of 4-day-old *dcl1-9* and *hyl1-2* mutants under continuous white light (30 μmol s^−1^ m^−2^), and we found that the hypocotyl lengths of *dcl1-9* and *hyl1-2* mutants are similar to those of WT seedlings grown under white light (S12A and S12B Fig).

To further examine the function of DCL1 and HYL1 under red light, we generated *DCL1* and *HYL1* overexpressing lines in WT background under the control of the 35S promoter (*p35S*::*PIF4-YFP/Col*, *p35S*::*DCL1-YFP/Col* and *p35S*::*HYL1-YFP/Col*). High expression levels of these genes were confirmed in transgenic lines (S13 Fig). The seedlings of *p35S*::*PIF4-YFP*, *p35S*::*DCL1-YFP*, and *p35S*::*HYL1-YFP* had longer hypocotyls than those of WT seedlings grown under any red light intensity (0.1, 0.5, 1,and 10μmol s^−1^ m^−2^)(Fig 6A and 6B). Together, these results indicated that DCL1and HYL1 act as negative regulators for plant photomorphogenesis in the red light signaling pathway.

To determine the genetic relationship between PIF4 and miRNA processor DCL1 or HYL1, we generated a *pif4-2/hyl-2* double mutant, as the homozygous *dcl1-9* line is sterile [26]. We found the *pif4-2/hyl-2* double mutant has a shorter hypocotyl length than those of *hyl1-2* and *pif4-2* single mutants grown under red light (Fig 6A and 6B), indicating that *HYL1* mutation can enhance the photomorphogenesis phenotype of the *pif4-2* mutant grown under red light; therefore, HYL1 and PIF4 have a synergistic function in the regulation of hypocotyl elongation. To investigate the genetic interaction of phyB and HYL1, we generated a *phyB/hyl1-2* double mutant. The hypocotyl length of *phyB/hyl1-2* mutant is shorter than that of *phyB* mutant and longer than that of *hyl1-2* single mutant (Fig 6A and 6B) grown under red light, demonstrating that *HYL1* acts genetically downstream of *PHYB* in the regulation of plant photomorphogenesis.

### MiRNAs are positive or negative regulators of plant photomorphogenesis

**Fig 7:**
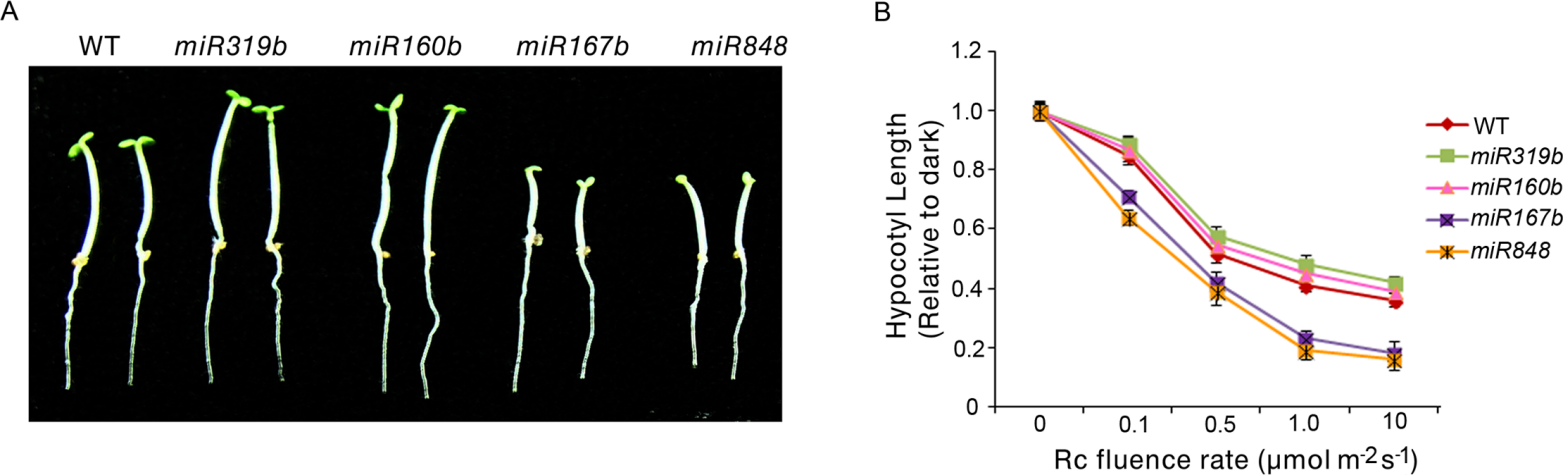
The roles of mature miRNAs in plant photomorphogenesis. **(A)** Visual phenotypes of WT, *miR319b*, *miR160b*, *miR167b* and *miR848* seedlings grown under red light (10 μmolm^-2^s^-1^) for 4 days. **(B)** Rc fluence rate response curves for hypocotyl lengths of WT, *miR319b*, *miR160b*, *miR167b* and *miR848* seedlings grown under red light (10 μmolm^-2^s^-1^) for 4 days. Data are means ± SEM of 30 plants. All the experiments have been performed for three biological replicates.

Because the microprocessors DCL1 and HYL1 regulate plant photomorphogenesis (Fig 6A and 6B), we speculated that mature miRNAs might also serve as regulators of hypocotyl growth. We examined the phenotypes of several mutants of miRNAs which are regulated by PIF4 (Fig 4; S1 and S2 Tables) and found that *miRNA319b* and *miRNA160b* mutants displayed longer hypocotyl phenotypes, while *miRNA167b* and *miRNA848* exhibited shorter hypocotyl phenotypes compared with WT seedlings grown under red light (Fig 7A and 7B). The hypocotyl lengths of these miRNA mutants were similar to those of WT seedlings grown under dark conditions (S11A and S11B Fig). Therefore, we concluded that mature miRNAs, including miRNA319b, miRNA160b, miRNA167b, and miRNA848, regulate plant photomorphogenesis either positively or negatively.

## Discussion

In this study, we uncovered a crosstalk between the light signaling pathway and the miRNA biogenesis pathway in *Arabidopsis*. It was known that PIF4 plays a negative role in the phytochrome B signaling pathway under red light, [6] and a positive role in cell elongation [36]. We found that PIF4 integrates red light signaling and miRNA biogenesis through the regulation of miRNA transcription and processing by affecting DCL1 stability during dark/red-light transitions. In addition, we found that the miRNA processing enzyme DCL1 and a group of miRNAs play an important role in plant photomorphogenesis.

**Fig 8:**
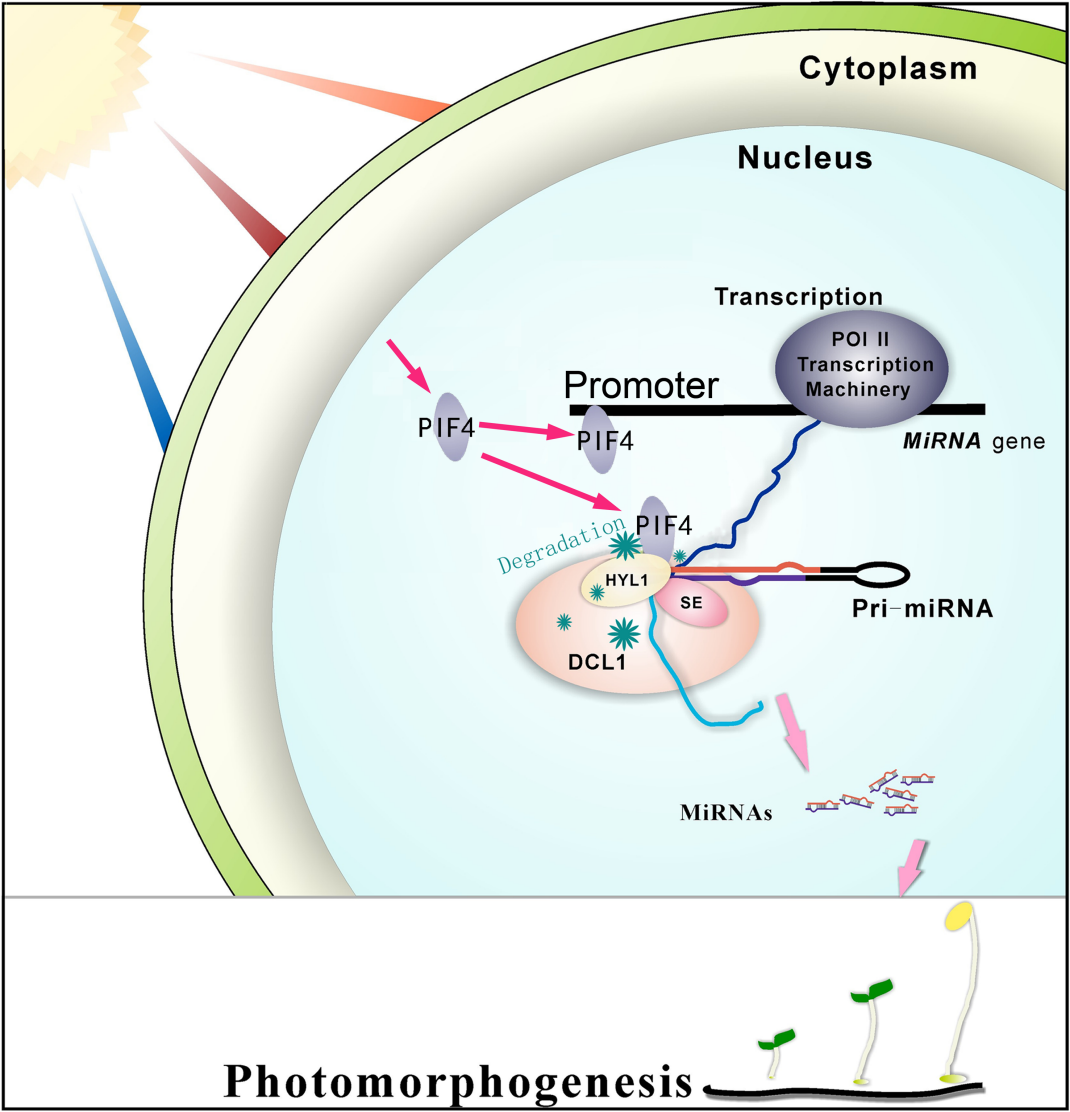
A working model for the roles of PIF4 in the transcription and processing of pri-miRNAs to regulate plant photomorphogenesis. PIF4, a factor involved in red light signaling, functions at both the transcription and processing levels to regulate the levels of some mature miRNAs which play positive or negative roles in plant photomorphogenesis. At the transcriptional level, PIF4 binds directly to the promoters of some miRNA genes and regulate their transcriptions. At the post-transcriptional level, PIF4 interacts with DCL1 and HYL1 to promote the destabilization of them during dark to red light transition and regulate the processing of primary miRNAs.

For the biogenesis of miRNAs, at least three steps are necessary: the transcription of primary miRNAs (pri-miRNAs) from miRNA genes; the processing of pri-miRNAs to precursor miRNAs (pre-miRNAs); and the processing of pre-miRNAs to mature miRNAs. It was known that the interaction of PIF4 with the photo-activated Pfr form of phyB modulates a subset of downstream factors through binding to the promoters of these genes [6]. In this study, we found that the transcription factor PIF4 functions at both the transcriptional and post-transcriptional levels to regulate miRNA biogenesis. At the transcriptional level, PIF4 can bind directly to the promoters of a group of miRNA genes and control their transcription (Fig 8). In addition, we noticed that the expressions of some miRNAs increase, while others decrease (Fig 5A), implying that PIF4 may serve as either a positive or negative transcription regulator for a miRNA gene. At post-transcriptional level, PIF4 interacts with DCL1 and HYL1 to promote the destabilization of these essential microprocessor proteins and regulate the levels of mature miRNAs during dark-to-red-light transition (Fig 8). The PIF4-dependent DCL1 degradation during dark-to-red-light transition might also provide a logical explanation for small RNA-seq data which show that the accumulation levels of most miRNAs decrease, whereas only a few miRNAs increase in plants grown under red light conditions (S6 Fig).

The light-dependent stabilization of HYL1 is maintained by the light signal negative factor COP1 [23]. Because the miRNA processing activity is impaired when DCL1 or HYL1 is degraded, plants might be able to adjust their levels of precisely processed miRNAs by modulating their DCL1 and HYL1 levels in response to different light conditions. A recent study reported that the levels of miR167, miR168, miR171, and miR398 increase by day and decrease by night, but the oscillatory pattern is not regulated by the circadian clock [37]. Therefore, the biological significance of DCL1 fluctuation can be inferred by the diurnal oscillation of the short-lived miRNAs.

We suspected that DCL1 is degraded by a protease or several proteases to adjust miRNA processing, similar to the report that HYL1 is destabilized by COP1 in response to light/dark transition. In animals where miRNA homeostasis including DICER1 and AGO2 is regulated by autophagy [23]. Future studies will focus on revealing if and how autophagy is involved in the regulation of DCL1 or HYL1 stability and identifying the protease(s) responsible for plant microprocessor degradation.

We identified the *miR167b* mutant which exhibited a shorter hypocotyl phenotype. One of the mi167b targets is ARF8, which inhibits hypocotyl elongation under red and blue light [38]; this supports that miR167b plays a role in plant photomorphogenesis. We found that miR167b and miR848 play negative roles, whereas miR319b and miR160b play positive roles in plant photomorphogenesis. Conversely, DCL1 or HYL1 is a negative regulator of photomorphogenesis. We speculated that the overall phenotypes of *dcl1* and *hyl1* mutants in photomorphogenesis result from the coordinated effects of the diverse functions of miRNAs in the regulation of hypocotyl growth.

Multiple environmental and hormonal signals for plant growth regulation in *Arabidopsis* are integrated by PIF4 [17,18]. In this study, we placed the microprocessor DCL1 and HYL1 as a critical node in the red light signaling pathways by binding them to PIF4, and further studies will reveal whether DCL1 also acts as a node in other signal pathways. Our previous results indicated that blue light signaling factor CDF2 is involved in miRNA biogenesis [23], and further studies will direct toward testing whether the destabilization of DCL1 is dependent on blue light and the potential crosstalk between blue light signaling and miRNA biogenesis.

## Materials and methods

### Plant materials and growth conditions

*Arabidopsis thaliana* (ecotype Col-0), *phyB* (SALK_022035C), *dcl1-9* [25,39], *hyl1-2* (Salk_064863), *pif4*-2 (CS66043), *miR160b* (SALK_152649), *miR319b* (SALK_037093C), *miR167b* (CS872594) and *miR848* (CS812734) mutants were used. All plants were grown in soil or Murashige and Skoog (MS) medium at 16 hr light/8 hr dark photoperiod unless specifically indicated otherwise.

### Constructs and transgenic plants

The PIF4, DCL1 and HYL1 coding sequences were cloned into pCambia1301 with the CaMV35S promoter and a YFP tag [25] and confirmed by sequencing. Primers used to amplify these genes are listed in S3 Table. The upstream regulatory sequence of *PIF4* and coding region were cloned into pCambia1301 with a YFP tag to generate pPIF4::PIF4-YFP vector and confirmed by sequencing. Primers are listed in S3 Table.

All binary vectors were introduced into *Agrobacterium tumefaciens* (strain GV3101) by electroporation. Plants were transformed by floral dip [40], and the transformats were selected on MS medium with hygromycin (50 mg/l). Lines containing a single T-DNA insertion were selected on the basis of the segregation ratio of the resistant and susceptible plants to hygromycin in the progeny of these primary transformats. Homozygous stocks were selected from these lines and at least 15 T_2_ independent lines were analyzed for each construct.

### Yeast two-hybrid screen

Yeast transformation and library screening were performed according to the Pro-Quest Two-Hybrid System Manual (Matchmaker user’s manual, Invitrogen). The experiments was performed as described in Sun et al. [21]. The size of F1 fragment of PIF4 was 309 aa (from aa 123 to aa 431), F2 fragment was 221 aa (from aa 211 to aa 431), F3 fragment was 177 aa (from aa 255 to aa 431) and F4 fragment was 119 aa (from aa 256 to aa 374).

### Pull-down assays

The cDNAs of *PIF4* and *DCL1* were amplified and subcloned to the pMAL-c2x and pGEX4T-1 plasmids, and confirmed by sequencing. Primers used to amplify the genes are listed in S3 Table. MBP-DCL1-RBD and MBP were expressed in *E*. *coli* BL21 (DE3) and purified according to the manufacturer’s protocol (New England Biolabs). GST-PIF4 and GST were purified using glutathione–agarose 4B (Peptron) beads. For protein pull-down assays, GST-PIF4 was incubated with the MBP-DCL1-RBD bound to amylose resin, mixed with total protein extracts in 1ml protein pull-down buffer (40 mM HEPES-KOH, pH 7.5, 10 mM KCl, 3 mM MgCl_2_, 0.4 M sucrose, 1 mM EDTA, 1 mM DTT, and 0.2% Triton X-100), and then incubated at 4°C for 1 h with agitation. GST and MBP proteins were used as negative controls in parallel assays. The beads were washed four times with the binding buffer. Proteins were eluted and further analyzed by immunoblotting using appropriate antibodies.

### Coimmunoprecipitation (Co-IP) assay

The lines of coexpressing *hemagglutinin (HA)-labeled PIF4* (*pPIF4*::*PIF4-HA)* and *YFP-labeled DCL1* (*pDCL1*::*DCL1-YFP*) were generated by crossing *pDCL1*::*DCL1-YFP*/*dcl1-9* [21] with *pPIF4*::*PIF4-HA*/*pif4-2* obtained by crossing *pPIF4*::*PIF4-HA*/Col with *pif4-2* mutant. For control lines, plants co-expressing *pPIF4*::*PIF4-HA/pif4-2* and *p35S*::*YFP*/Col were generated by transforming the *pPIF4*::*PIF4-HA/pif4-2* line with the *p35*::*YFP* construct. The homozygous lines were grown in MS medium for 4 days, then seedlings were ground in liquid nitrogen and homogenized in three volumes of extraction buffer (50 mM Tris-HCl at pH 8.0, 150 mM NaCl, 0.5% TritonX-100, 0.2% 2-mercaptoethanol, 5% glycerol) containing complete proteinase inhibitor cocktail (Roche) using a mortar and pestle set. Cell debris was pellet by centrifugation for 10 min at 13,000 g. The Co-IP experiments were performed using HA Tag IP/Co-IP Kit according to the manufacturer’s protocol (Thermo Pierce) and the previous report [21].

### Bimolecular fluorescence complementation (BiFC) assays

The coding sequence of PIF4 [41], DCL1, HYL1, DCL1-9, the N-terminal fragment of PIF4 (PIF4-N, from aa 1 to 254), and the C-terminal fragment of PIF4 (PIF4-C, from aa 255 to 431) were subcloned into pCambia1301 Vectors with YFPN or YFPC tag [25] and confirmed by sequencing. Primers used to amplify these genes are listed in S3 Table. Plasmid pairs were expressed in tobacco leaves, the relevant negative control was performed at the same time. 48 hours after co-inoculation, BiFC signals were visualized with a DeltaVision Personal DV system (Applied Precision) using an Olympus UPLANAPO water immersion objective lens (60×/1.20 numerical aperture) [42].

### Cell-free degradation assay and chemical treatments

Degradation assay was performed as previous reported with some modifications [43], 4-day-old seedlings of wild-type (18h light/6-h dark photoperiod) were ground in liquid nitrogen and resuspended in a buffer (25 mM Tris pH 7.5, 10 mM MgCl_2_, 5 mM DTT, 10 mM NaCl and 10 mM ATP). Cell debris was pellet by centrifugation and equal amounts of extract were transferred to individual tubes, which were incubated at room temperature for 2 h, and reactions were stopped by adding an equal volume of 2×protein gel-loading buffer. Equal amounts of sample were then analyzed by western blots with an anti-DCL1 antibody and an anti-ACTIN antibody.

For chemical treatments, 4-day-old seedlings of WT (18h light/6-h dark photoperiod) were treated with protease inhibitor cocktail tablets (PIs, Roche), E64D (Sigma), 3-MA (Sigma) or BTH (Sigma) at the indicated concentrations. Blots were detected with an anti-DCL1 antibody and an anti-ACTIN antibody.

### Real-time quantitative PCR

Real-time quantitative PCR assays were performed as previously described [21]. Briefly, the first strand cDNA was synthesized from the total RNA (1 μg) with M-MLV reverse transcriptase (Promega) and used as the template for subsequent PCR amplification. The real-time quantitative PCR (RT-qPCR) for examination of pri-miRNA expression was carried out with a BIO-RAD CFX^TM^ Real-Time System. The *ACTIN* gene was used as an internal control for normalization of the cDNA template. Each PCR was repeated at least three times. The data were analyzed with a Bio-Rad iCycler iQ Real-Time Detection System. The expressions of genes were calculated using the relative 2^–ΔΔCt^ method [44].

### Chromatin immunoprecipitation (ChIP)

The ChIP assay was performed as described [45] using 4-d-old seedlings. Transgenic seedlings harboring *pPIF4*::*PIF4-YFP* were harvested in cross-linking buffer (0.4M sucrose, 10mM Tris-HCl (pH8.0), 1mM PMSF, ImM EDTA, 1% formaldehyde) for 10 min using vacuum infiltration. The cross-linking was stopped in 2M glycine. After chromatin shearing, about 5 μg anti-GFP monoclonal antibody (Sigma, G6495) was added to the samples and incubated at 4°C overnight. Beads were then washed and eluted with the lysis buffer (0.1M NaHCO_3_, 1%SDS). DNA was precipitated using ethanol, and resuspended in 50 μl water after reversing cross-linking. The immunoprecipitated genomic DNA were used for PCR and quantified by real-time PCR. *ACTIN* gene was used as an internal control. Primers used to amplify the promoters of some miRNA genes are listed in S3 Table.

### DNA electrophoretic mobility shift assay (DNA EMSA)

The EMSA was performed as described previously [46] with modification. The sense and antisence sequences of miR160 promoter (S3 Table) were synthesized and the sense sequence was 3’end-labeled with biotin. The promoter fragment synthesized has a G-box DNA-sequence motif (CACGTG), a variant of the canonical E-box motif (CANNTG) which was known to be the binding site of PIFs [32]. The single-strand DNAs were mixed, denatured at 95°C for 5 min, and slowly cooled to room temperature to form the double-strand DNAs. The unlabeled double-strand DNA with the same sequence was used as a competitor. Using Light Shift Chemiluminescent EMSA Kit (Thermo Pierce), EMSA assays were performed in 20 μl reaction buffer according to manufactory’s protocol. The mixtures were incubated on ice for 30 min and then fractioned on polyacrylamide gels in 1×TBE buffer for about 60 min. The gels were transferred to a nylon membrane (GE Healthcare) and then the biotin-labeled oligonucletides were detected by Chemiluminescence (Thermo Pierce).

### Histochemical GUS staining

GUS staining was performed according to the standard procedure [47] with a modified buffer (1mg/ml 5-bromo-4-chloro-3-indolyl-b-D-glucuronic acid cyclohexylammonium salt, 50 mM sodium phosphate, pH 7.0, 0.1% Triton X-100, 2 mM potassium ferrocyanide, 2 mM potassium ferricyanide, and 10 mM EDTA). Plant tissues were incubated in the buffer at 37°C in the dark overnight, and then cleared with 75% ethanol, followed by observation [21].

### Hypocotyl growth analyses

Light treatment of seedlings was performed as described [48] with some modifications. Seedlings of each line were grown on the same 100 mm Petri dish for each repeat. Seeds were sterilized by soaking for 5 min in 70% ethanol, followed by 5 min in 95% ethanol. The seeds were vernalizated for 3 days at 4°C in the dark. Plates were placed into white light for 3 h to induce germination and placed in a growth chamber at 21°C in darkness for 21 h before being transferred to the experimental light conditions. Seedlings were moved to red light (0.1, 0.5, 1 and 10 μmol s^-1^ m^-2^) and white light (10 μmol s^-1^ m^-2^) growth chambers (Percival Scientific Inc., Perry, Iowa, USA) at 21°C for 4 days, respectively. Dark control seedlings were kept in darkness. Hypocotyl lengths of at least 30 seedlings were measured after the light treatments.

### Small RNA gel blot

Small RNAs were isolated from seedlings of 4-day-old plants using *mir*Vana™miRNA Isolation Kit (Ambion, AM1561). Small RNA about three micrograms was fractionated on a 15% polyacrylamide gel containing 8M urea, and then transferred to a nylon transfer membrane (GE Healthcare). The antisense oligonucletides (S3 Table) were synthesized and 3’end-labeled as probes with biotin. Hybridization was performed overnight at 42°C in hybridization buffer (Ambion, AM8663). A probe complementary to U6 (5’CATCCTTGCGCAGGGG CCA 3’) was used as a loading control.

### Small RNA deep sequencing

RT-qPCR assays were performed as previously described [21]. Small RNAs were isolated from seedlings of 4-day-old plants using mirVana™miRNA Isolation Kit (Ambion, AM1561) and sequenced by Illumina Solexa high-throughput sequencing. The sequencing data for all known miRNAs were subjected to hierarchical clustering in an unsupervised manner to analyze the extent of differential miRNA expression [31].

## Supporting information

S1 Fig Characterization of *pPIF4*::*PIF4-HA/*Col, *p35S*::*PIF4-N/pif4-2* and *p35S*::*PIF4-C/pif4-2 Arabidopsis* transgenic lines. (A) The relative expression levels of *PIF4* in *pPIF4*::*PIF4-HA/*Col seedlings compared to *PIF4* in WT seedlings. (B) The relative expression levels of *PIF4-N* in *p35S*::*PIF4-N/pif4-2* seedlings compared to *PIF4* in WT seedlings. (C) The relative expression levels of *PIF4-C* in *p35S*::*PIF4-C/pif4-2* seedlings compared to *PIF4* in WT seedlings. Data are given as means ± SD. All the experiments have been performed for three biological replicates.
(TIF)

S2 Fig Yeast transactivation activity assays show that the C terminal fragment of PIF4 activated transcription. GAL4-BD, GAL4 DNA-binding domain; GAL4-AD, GAL4 activation domain; NLS, Nuclear localization signal; MCS, multiple cloning site; CDS, coding sequence of PIF4; APB, active phytochrome binding motif; bHLH domain, basic helix-loop-helix domain.
(TIF)

S3 Fig A biological replicate of the experiments related to Fig 2. (A) Anti-GFP western blots of *pPIF4*::*PIF4-YFP*/Col seedlings grown in continuous darkness for 4 d and then transferred to red light condition for 3 or 5 h. (B) Anti-DCL1 western blots of WT and *pif4-2* mutant seedlings grown in red light for 4 d followed by transferring to dark condition for 3 or 5 h (red light to dark), or grown in continuous darkness for 4 d followed by transferring to red light for 3 or 5 h (dark to red light). (C) Anti-DCL1 western blots of *p35S*::*PIF4-N*/*pif4-2*, *p35S*::*PIF4-C*/*pif4-2* seedlings grown in red light for 4 d and then transferred to dark condition for 3 and 5 h.
(TIF)

S4 Fig A biological replicate of the experiments related to Fig 2. (A) Anti-GFP western blots of *pPIF4*::*PIF4-YFP*/Col seedlings grown in continuous darkness for 4 d and then transferred to red light condition for 3 or 5 h. (B) Anti-DCL1 western blots of WT and *pif4-2* mutant seedlings grown in red light for 4 d followed by transferring to dark condition for 3 or 5 h (red light to dark), or grown in continuous darkness for 4 d followed by transferring to red light for 3 or 5 h (dark to red light). (C) Anti-DCL1 western blots of *p35S*::*PIF4-N*/*pif4-2*, *p35S*::*PIF4-C*/*pif4-2* seedlings grown in red light for 4 d and then transferred to dark condition for 3 and 5 h.
(TIF)

S5 Fig Anti-GFP Western blots of *pDCL1*::*DCL1*-YFP transgenic line grown in continuous darkness for 4 d and then transferred to red light condition for 2, 4, 6, 8 h. All the western blots have been performed for three biological replicates.
(TIF)

S6 Fig HYL1 is destabilized by PIF4 during dark to red light transition. Anti-HYL1 western blots of WT and *pif4-2* mutant seedlings grown in red light for 4 d followed by transferring to dark condition for 3 or 5 h (red light to dark), or grown in continuous darkness for 4 d followed by transferring to red light for 3 or 5 h (dark to red light). All the experiments have been performed for three biological replicates with similar results.
(TIF)

S7 Fig The transcript levels of *DCL1* and *HYL1* in *pif4-2* mutant compared to those in WT. (A) The *DCL1* transcript levels were examined by real-time PCR in WT, *pif4-2* grown under red light. (B) The *HYL1* transcript levels were examined by real-time PCR in WT, *pif4-2* grown under red light. Four-day-old dark-grown seedlings were illuminated with red light for 0, 1, 2, 3, 4, 5, 6, 8 h before tissues were collected for RNA extraction. Data are given as means ± SD. All the experiments have been performed for three biological replicates.
(TIF)

S8 Fig Heat map of miRNA differential expression in WT seedlings treated with red light compared to WT seedlings in dark. Four-day of dark grown seedlings were illuminated with red light for 2h and 8h before tissues were collected for RNA extraction. Small RNAs were isolated and sequenced by Solexa high-throughput sequencing.
(TIF)

S9 Fig MiRNAs differentially expressed between WT and *pif4-2* or WT and *hyl1-2* mutant. (A) Comparison of miRNAs differentially expressed between WT and *pif4-2* mutant. (B) Comparison of miRNAs differentially expressed between WT and *hyl1-2* mutant. The seedlings were illuminated with red light for 4-days before tissues were collected for RNA extraction. Small RNAs were isolated and sequenced by Solexa high-throughput sequencing.
(TIF)

S10 Fig DNA EMSAs show the interaction between PIF4 and the promoter of miR160b. Each lane was added 1 nM of labeled fragment of miR160b promoter and purified recombinant proteins indicated, including PIF4-MBP or MBP. Unlabeled fragment of miR160b promoter was used as a competitive probe (lane 8). All the experiments have been performed for three biological replicates with similar results.
(TIF)

S11 Fig Hypocotyl growths of indicated seedlings grown in dark. (A) Visual phenotypes of indicated seedlings grown in dark. (B) The hypocotyl lengths of seedlings in (A). Data are means ± SEM of 30 plants. All the experiments have been performed for three biological replicates.
(TIF)

S12 Fig Phenotypes of *DCL1* and *HYL1* mutants and overexpression lines. (A) Visual phenotypes of indicated seedlings grown under white light. (B) The hypocotyl lengths of seedlings in (A). Data are means ± SEM of 30 plants. All the experiments have been performed for three biological replicates.
(TIF)

S13 Fig The relative expression levels of *DCL1* in *p35S*::*DCL1-YFP*, *HYL1* in *p35S*::*HYL1-YFP*, and *FIF4* in *p35S*::*PIF4-YFP* seedlings compared to the corresponding gene in WT seedlings. Data are given as means ± SD. All the experiments have been performed for three biological replicates.
(TIF)

S1 Table The different expression levels of miRNAs between WT and *pif4*. (XLSX)

S2 Table The different expression levels of miRNAs between WT and *hyl1-2*. (XLSX)

S3 Table Oligonucleotide primer sequences used in this study. (XLSX)
