# Supplementary figures and images for: Coordinated regulation of Arabidopsis microRNA biogenesis and red light signaling through Dicer-like 1 and phytochrome-interacting factor 4
Source: PLoS Genet. 2018 Mar 9;14(3):e1007247. doi: 10.1371/journal.pgen.1007247 (PMC5862502; doi:10.1371/journal.pgen.1007247)

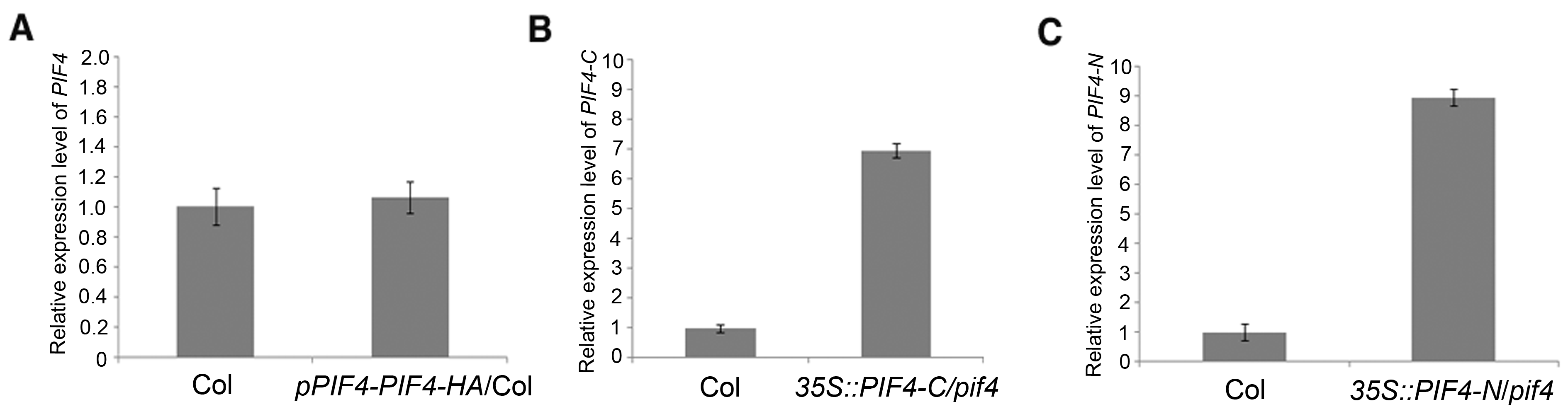

Supplement: S1 Fig — (A) The relative expression levels of PIF4 in pPIF4::PIF4-HA/Col seedlings compared to PIF4 in WT seedlings. (B) The relative expression levels of PIF4-N in p35S::PIF4-N/pif4-2 seedlings compared to PIF4 in WT seedlings. (C) The relative expression levels of PIF4-C in p35S::PIF4-C/pif4-2 seedlings compared to PIF4 in WT seedlings. Data are given as means ± SD. All the experiments have been performed for three biological replicates. (TIF) [file pgen.1007247.s001.tif]

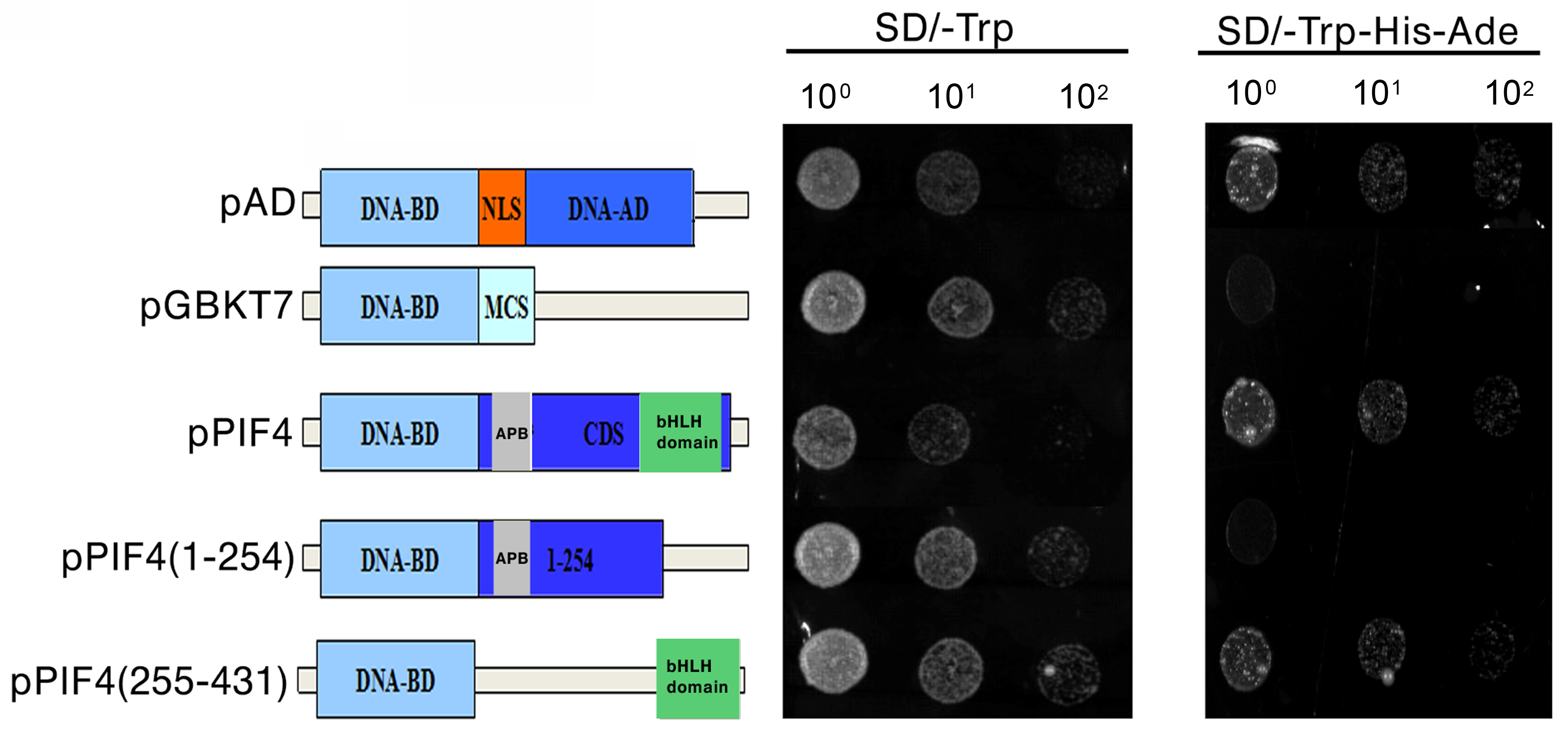

Supplement: S2 Fig — GAL4-BD, GAL4 DNA-binding domain; GAL4-AD, GAL4 activation domain; NLS, Nuclear localization signal; MCS, multiple cloning site; CDS, coding sequence of PIF4; APB, active phytochrome binding motif; bHLH domain, basic helix-loop-helix domain. (TIF) [file pgen.1007247.s002.tif]

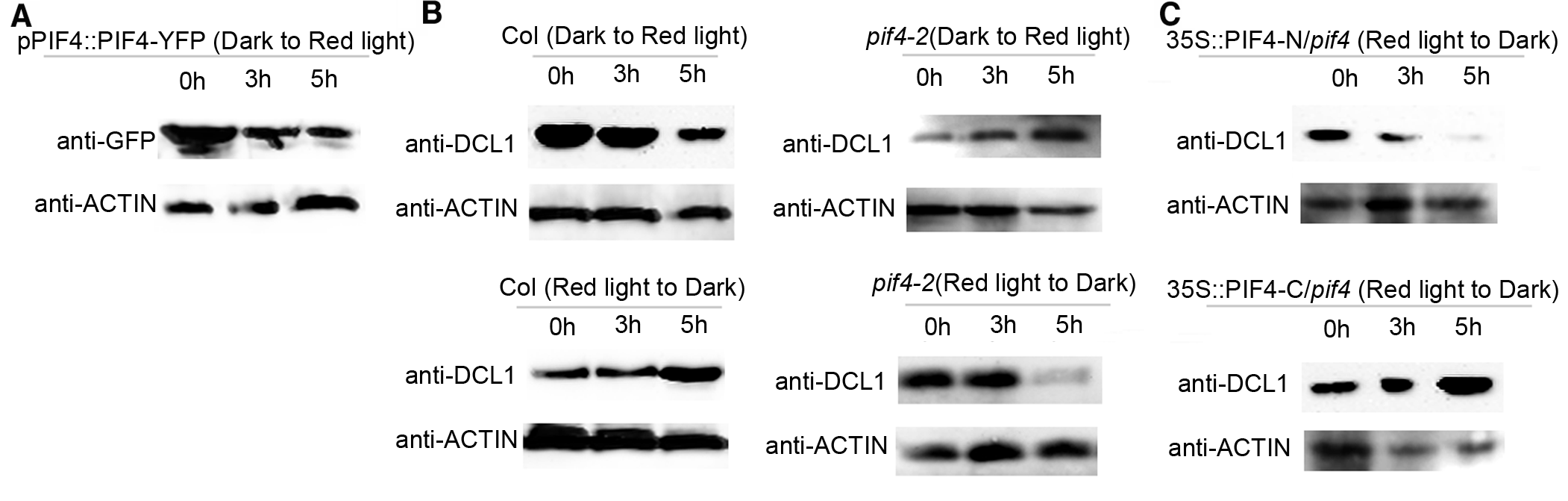

Supplement: S3 Fig — (A) Anti-GFP western blots of pPIF4::PIF4-YFP/Col seedlings grown in continuous darkness for 4 d and then transferred to red light condition for 3 or 5 h. (B) Anti-DCL1 western blots of WT and pif4-2 mutant seedlings grown in red light for 4 d followed by transferring to dark condition for 3 or 5 h (red light to dark), or grown in continuous darkness for 4 d followed by transferring to red light for 3 or 5 h (dark to red light). (C) Anti-DCL1 western blots of p35S::PIF4-N/pif4-2, p35S::PIF4-C/pif4-2 seedlings grown in red light for 4 d and then transferred to dark condition for 3 and 5 h. (TIF) [file pgen.1007247.s003.tif]

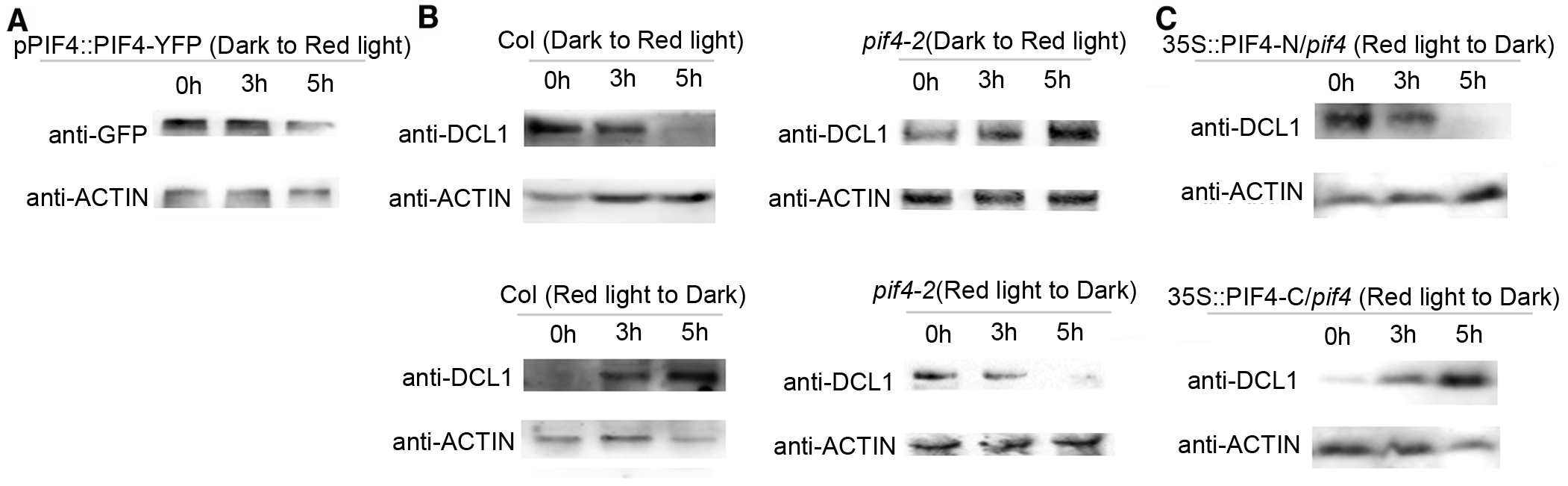

Supplement: S4 Fig — (A) Anti-GFP western blots of pPIF4::PIF4-YFP/Col seedlings grown in continuous darkness for 4 d and then transferred to red light condition for 3 or 5 h. (B) Anti-DCL1 western blots of WT and pif4-2 mutant seedlings grown in red light for 4 d followed by transferring to dark condition for 3 or 5 h (red light to dark), or grown in continuous darkness for 4 d followed by transferring to red light for 3 or 5 h (dark to red light). (C) Anti-DCL1 western blots of p35S::PIF4-N/pif4-2, p35S::PIF4-C/pif4-2 seedlings grown in red light for 4 d and then transferred to dark condition for 3 and 5 h. (TIF) [file pgen.1007247.s004.tif]

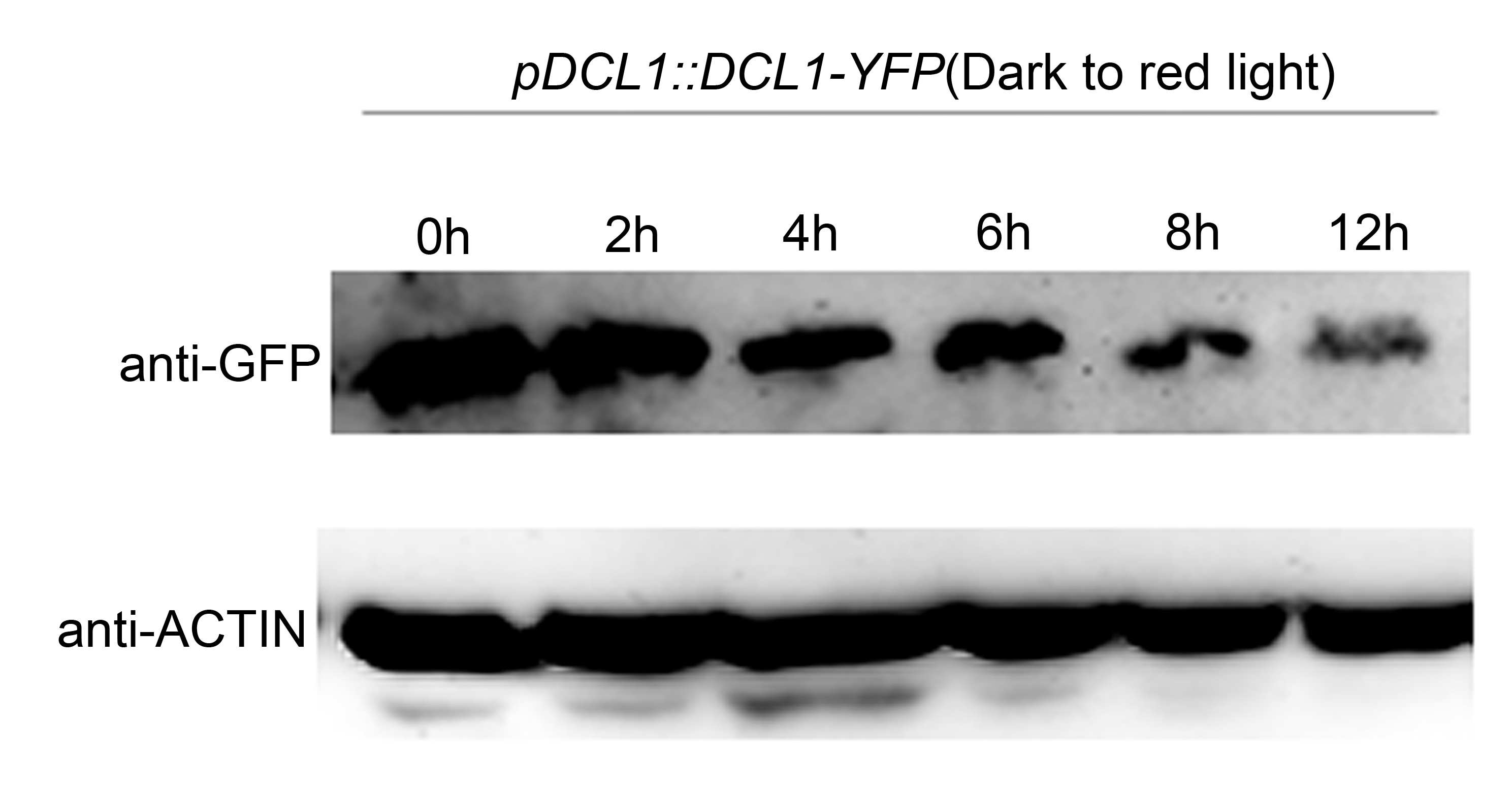

Supplement: S5 Fig — All the western blots have been performed for three biological replicates. (TIF) [file pgen.1007247.s005.tif]

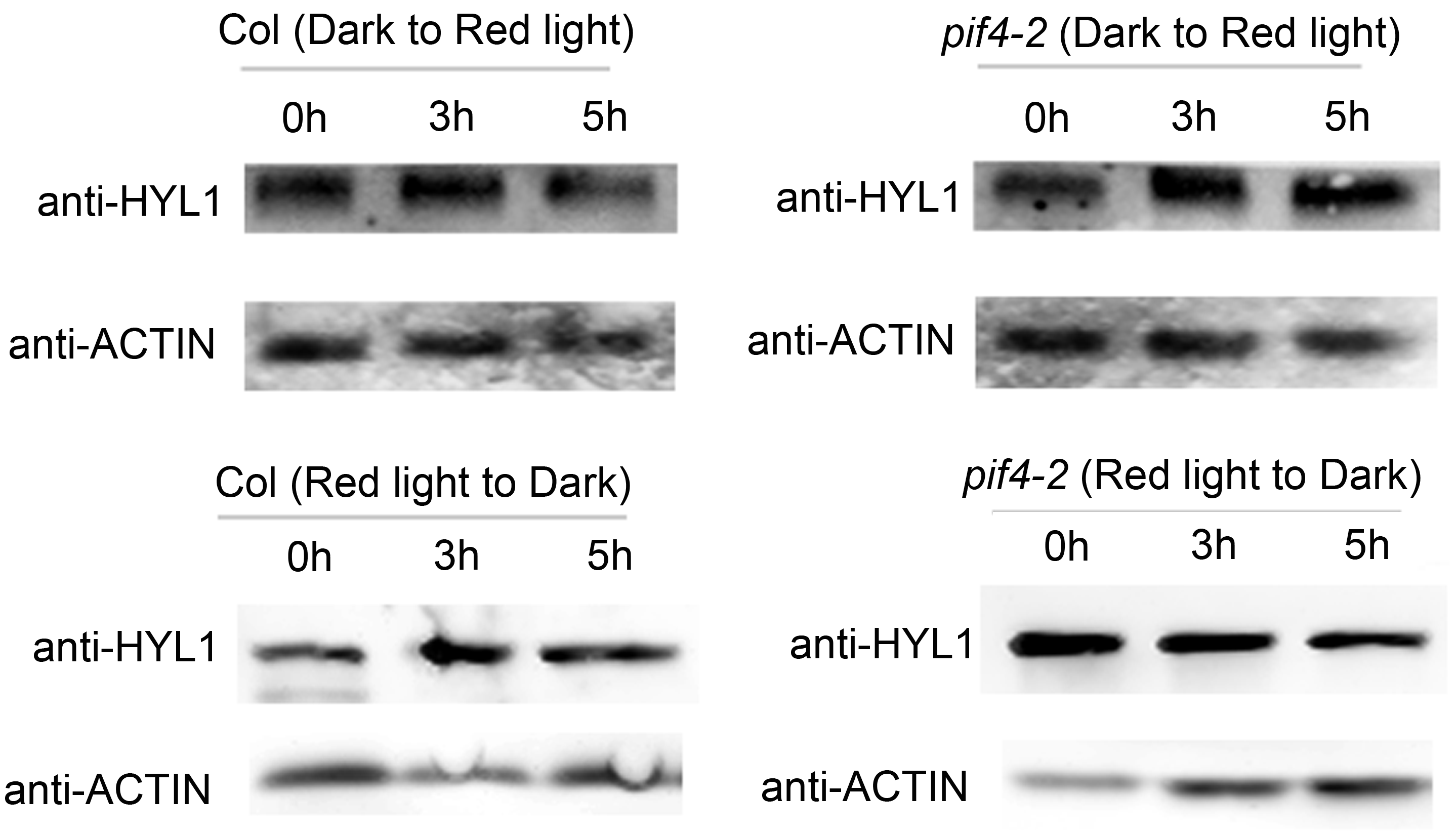

Supplement: S6 Fig — Anti-HYL1 western blots of WT and pif4-2 mutant seedlings grown in red light for 4 d followed by transferring to dark condition for 3 or 5 h (red light to dark), or grown in continuous darkness for 4 d followed by transferring to red light for 3 or 5 h (dark to red light). All the experiments have been performed for three biological replicates with similar results. (TIF) [file pgen.1007247.s006.tif]

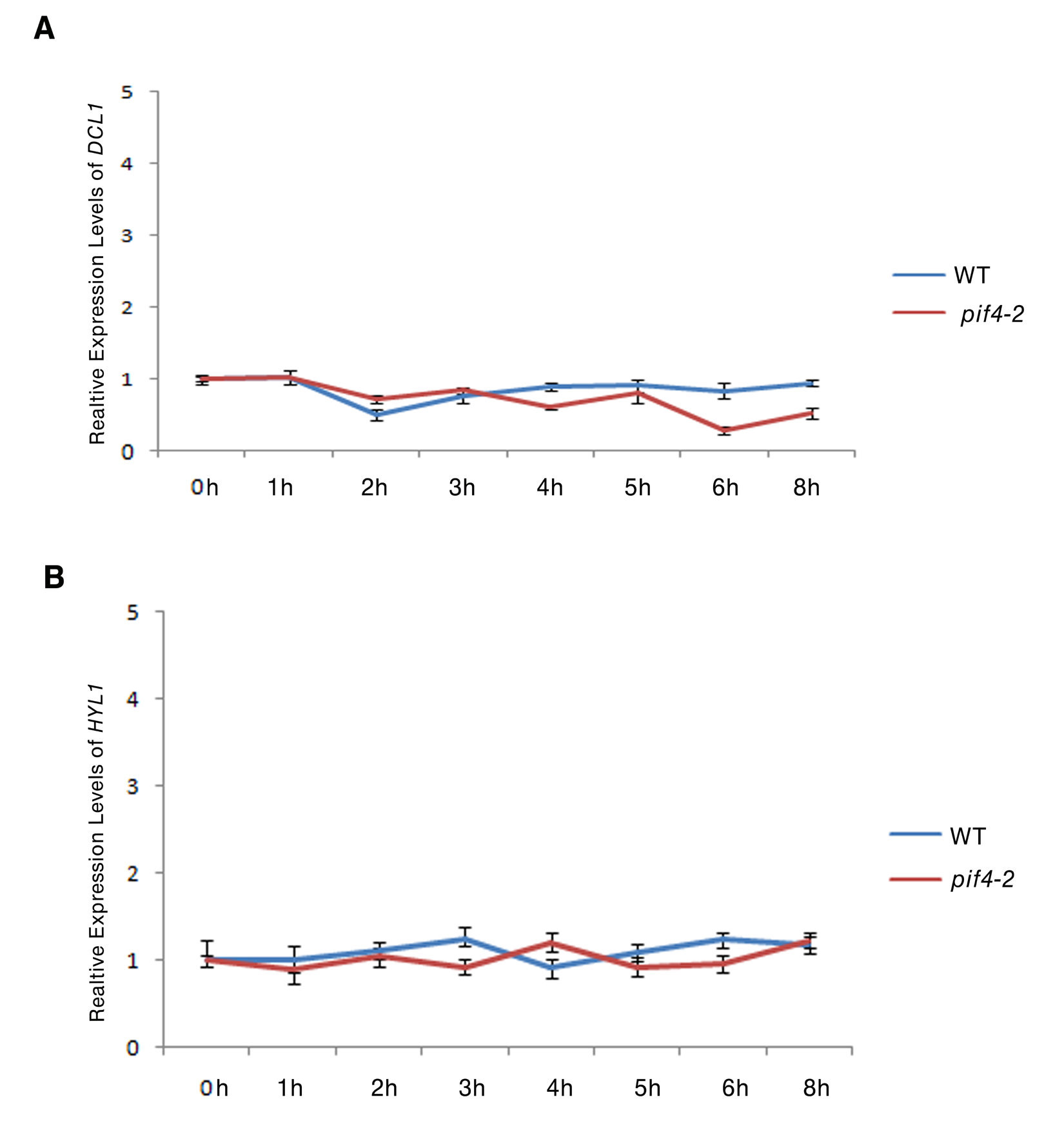

Supplement: S7 Fig — (A) The DCL1 transcript levels were examined by real-time PCR in WT, pif4-2 grown under red light. (B) The HYL1 transcript levels were examined by real-time PCR in WT, pif4-2 grown under red light. Four-day-old dark-grown seedlings were illuminated with red light for 0, 1, 2, 3, 4, 5, 6, 8 h before tissues were collected for RNA extraction. Data are given as means ± SD. All the experiments have been performed for three biological replicates. (TIF) [file pgen.1007247.s007.tif]

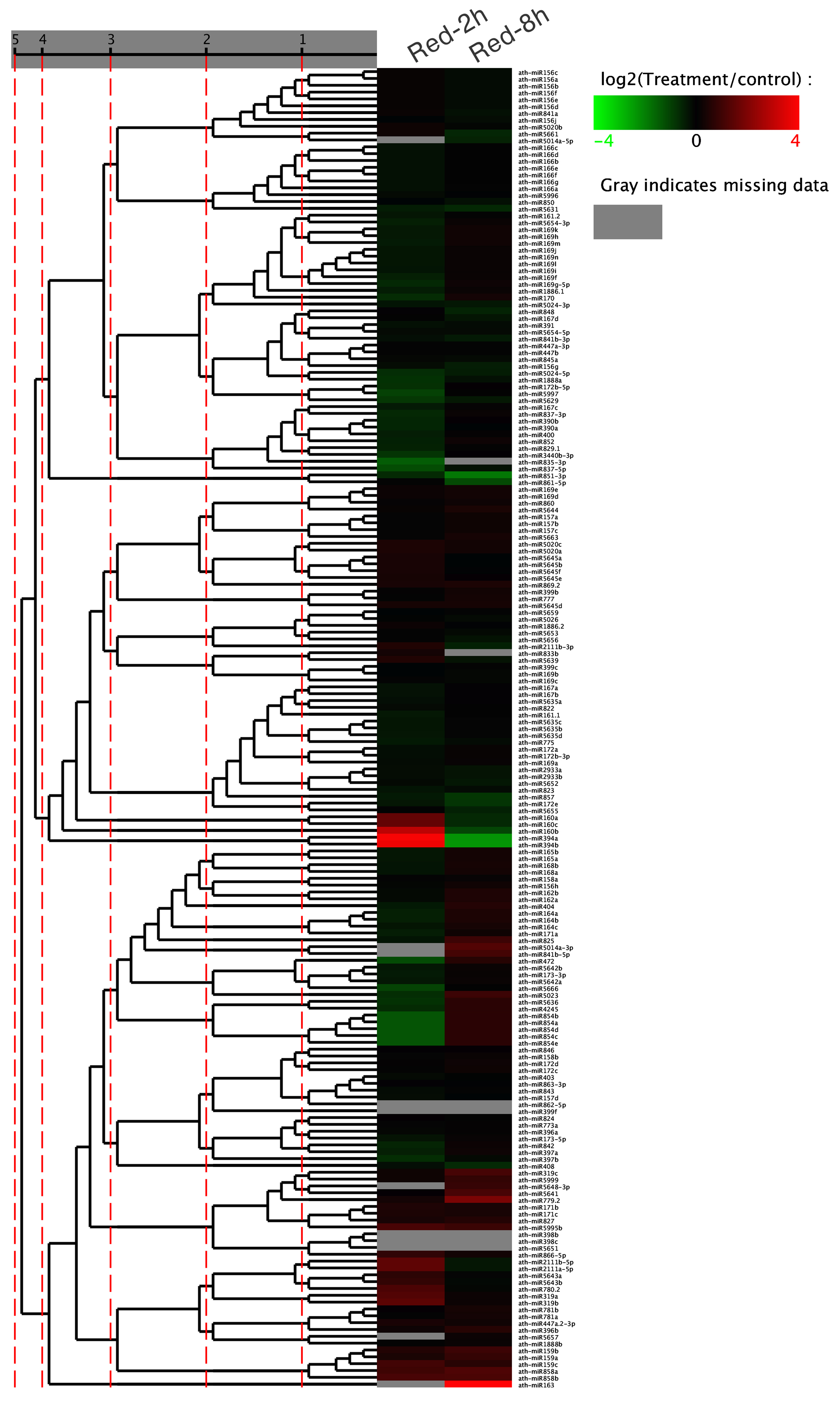

Supplement: S8 Fig — Four-day of dark grown seedlings were illuminated with red light for 2h and 8h before tissues were collected for RNA extraction. Small RNAs were isolated and sequenced by Solexa high-throughput sequencing. (TIF) [file pgen.1007247.s008.tif]

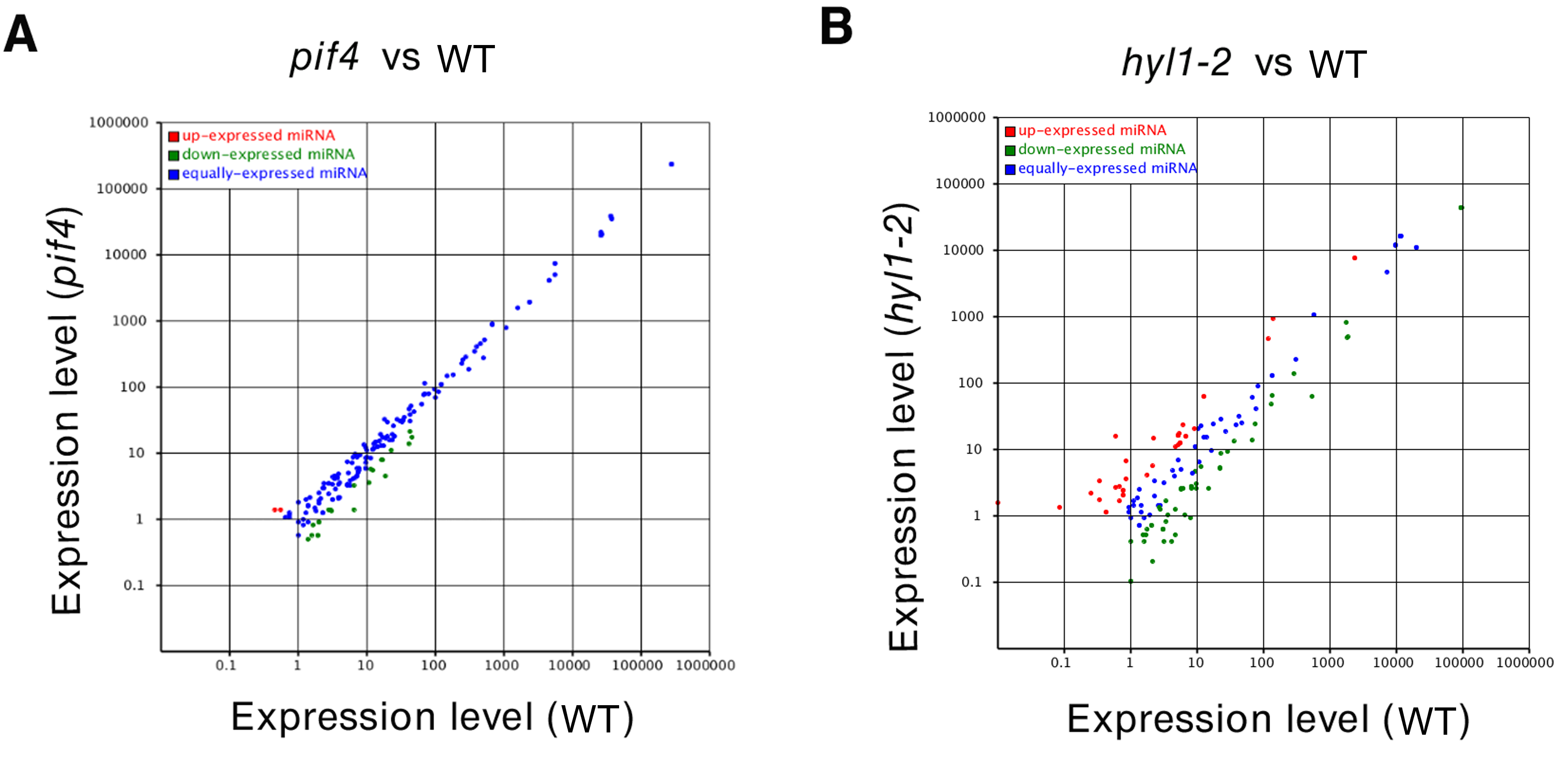

Supplement: S9 Fig — (A) Comparison of miRNAs differentially expressed between WT and pif4-2 mutant. (B) Comparison of miRNAs differentially expressed between WT and hyl1-2 mutant. The seedlings were illuminated with red light for 4-days before tissues were collected for RNA extraction. Small RNAs were isolated and sequenced by Solexa high-throughput sequencing. (TIF) [file pgen.1007247.s009.tif]

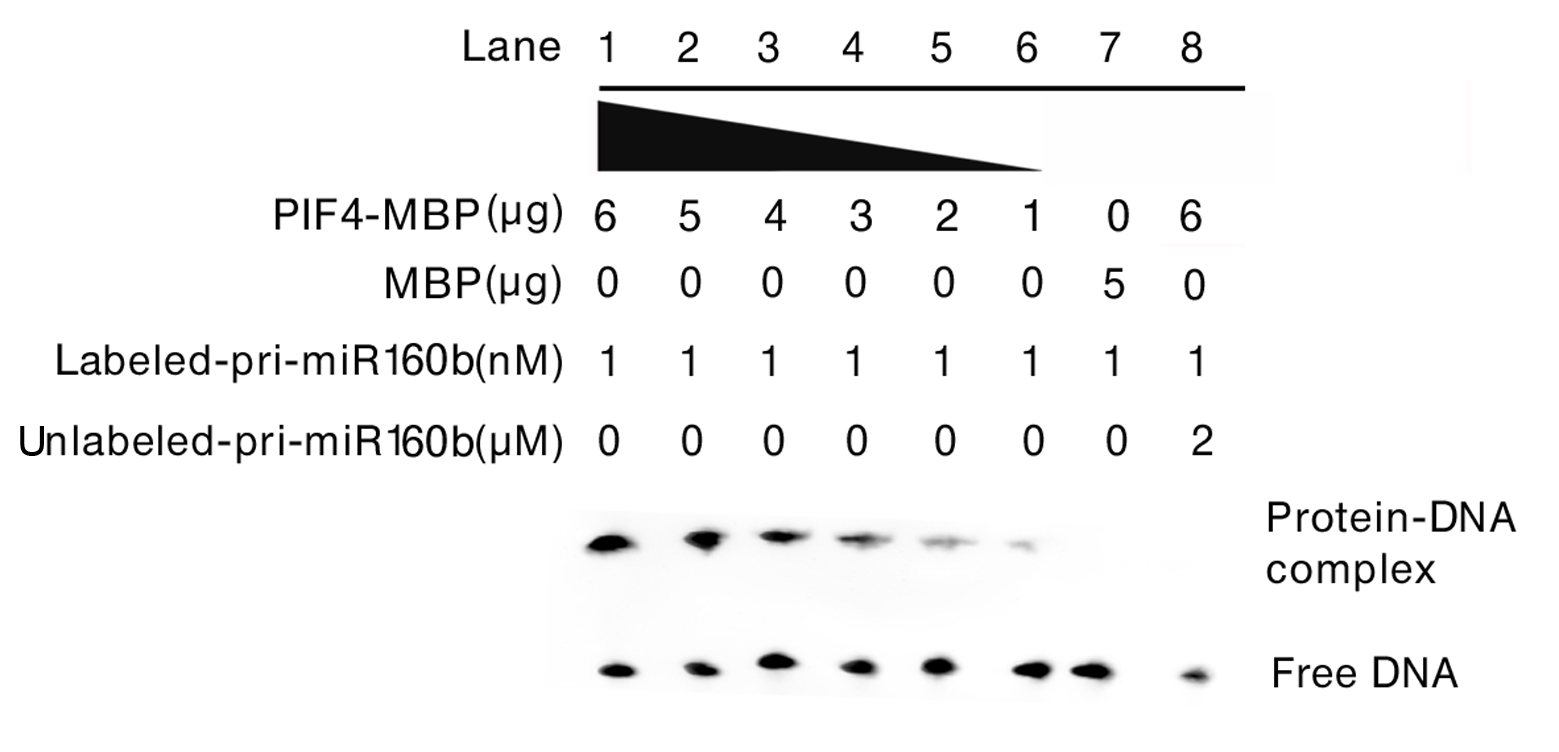

Supplement: S10 Fig — Each lane was added 1 nM of labeled fragment of miR160b promoter and purified recombinant proteins indicated, including PIF4-MBP or MBP. Unlabeled fragment of miR160b promoter was used as a competitive probe (lane 8). All the experiments have been performed for three biological replicates with similar results. (TIF) [file pgen.1007247.s010.tif]

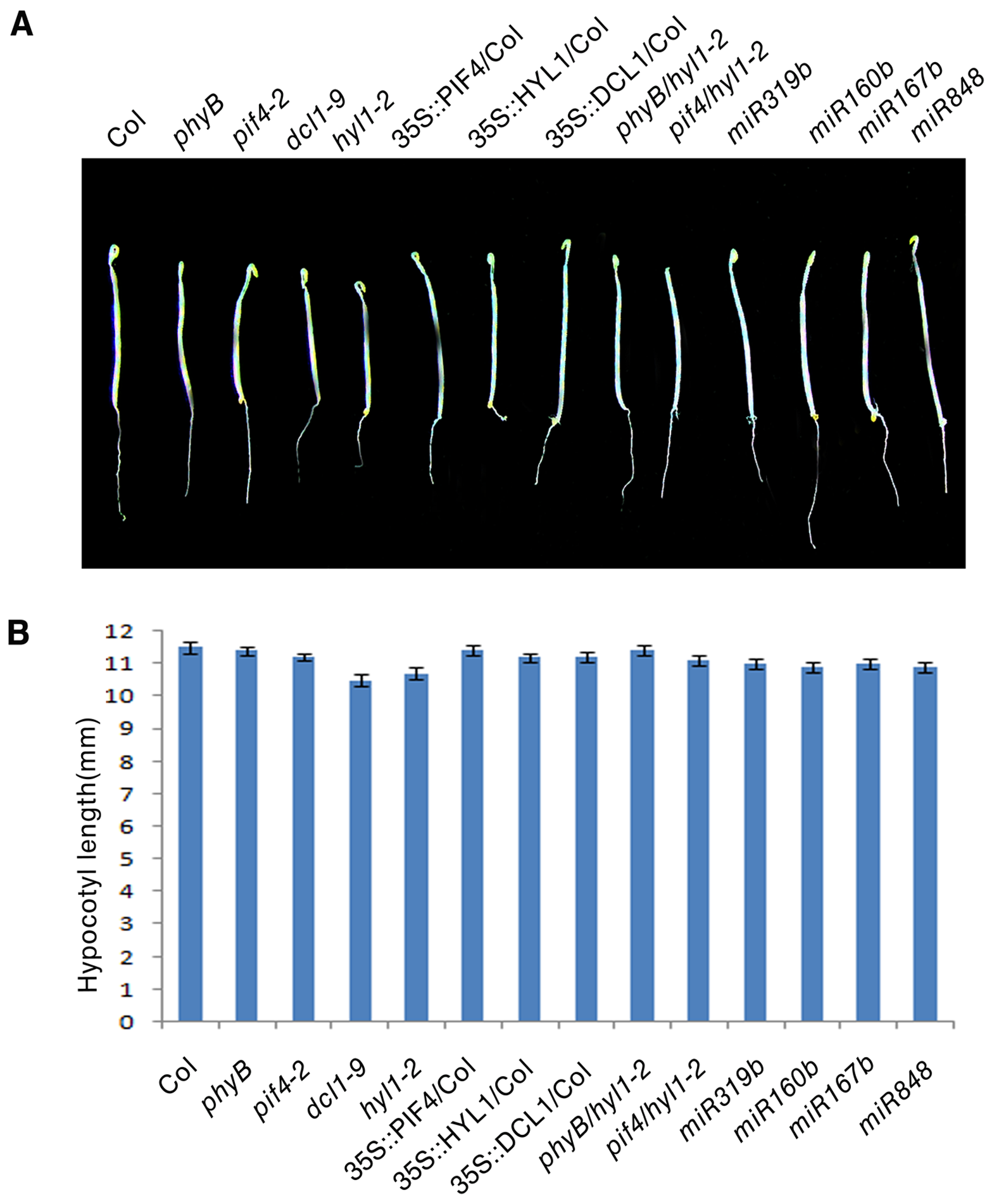

Supplement: S11 Fig — (A) Visual phenotypes of indicated seedlings grown in dark. (B) The hypocotyl lengths of seedlings in (A). Data are means ± SEM of 30 plants. All the experiments have been performed for three biological replicates. (TIF) [file pgen.1007247.s011.tif]

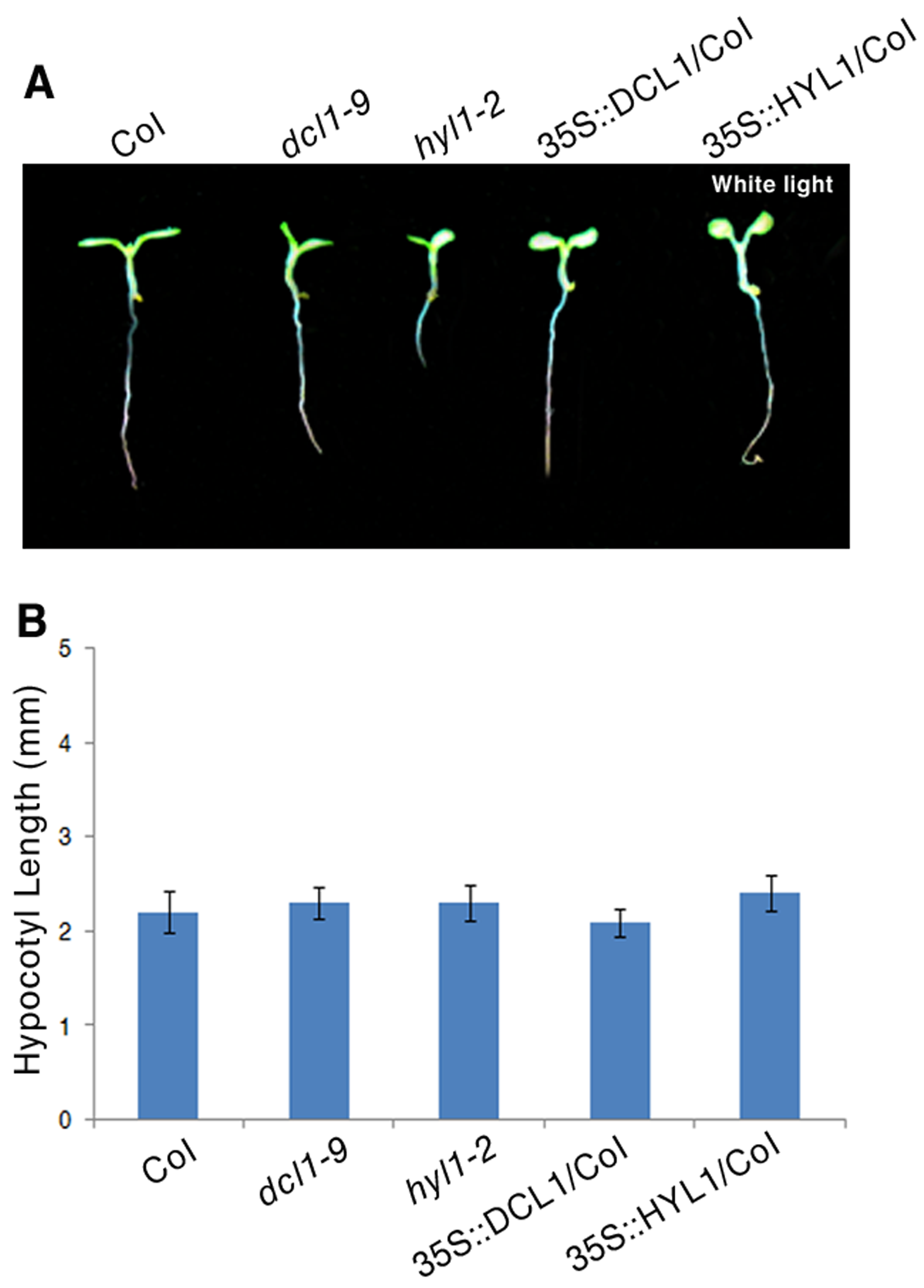

Supplement: S12 Fig — (A) Visual phenotypes of indicated seedlings grown under white light. (B) The hypocotyl lengths of seedlings in (A). Data are means ± SEM of 30 plants. All the experiments have been performed for three biological replicates. (TIF) [file pgen.1007247.s012.tif]

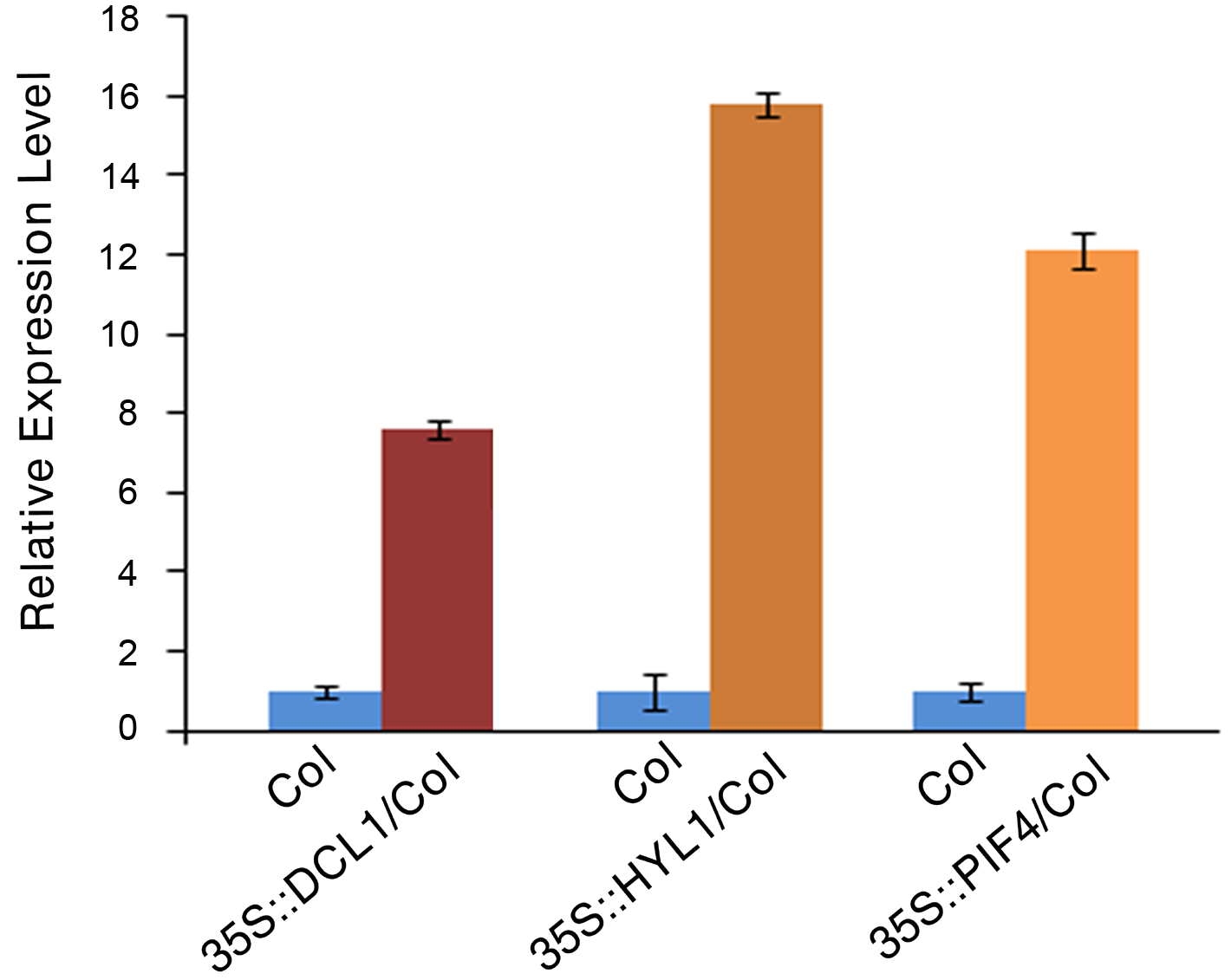

Supplement: S13 Fig — Data are given as means ± SD. All the experiments have been performed for three biological replicates. (TIF) [file pgen.1007247.s013.tif]
